# Olfactory bulb astrocytes mediate sensory circuit processing through Sox9 in the mouse brain

**DOI:** 10.1038/s41467-021-25444-3

**Published:** 2021-09-01

**Authors:** Kevin Ung, Teng-Wei Huang, Brittney Lozzi, Junsung Woo, Elizabeth Hanson, Brandon Pekarek, Burak Tepe, Debosmita Sardar, Yi-Ting Cheng, Gary Liu, Benjamin Deneen, Benjamin R. Arenkiel

**Affiliations:** 1 Program in Developmental Biology, Baylor College of Medicine, Houston, TX USA; 2 Center for Cell and Gene Therapy, Baylor College of Medicine, Houston, TX USA; 3 Department of Molecular and Human Genetics, Baylor College of Medicine, Houston, TX USA; 4 Medical Scientist Training Program, Baylor College of Medicine, Houston, TX USA; 5 Department of Neuroscience, Baylor College of Medicine, Houston, TX USA; 6 Department of Neurosurgery, Baylor College of Medicine, Houston, TX USA; 7 Jan and Dan Duncan Neurological Research Institute at Texas Children’s Hospital, Houston, TX USA

**Keywords:** Astrocyte, Molecular neuroscience, Olfactory bulb, Sensory processing

## Abstract

The role of transcription factors during astrocyte development and their subsequent effects on neuronal development has been well studied. Less is known about astrocytes contributions towards circuits and behavior in the adult brain. Astrocytes play important roles in synaptic development and modulation, however their contributions towards neuronal sensory function and maintenance of neuronal circuit architecture remain unclear. Here, we show that loss of the transcription factor *Sox9* results in both anatomical and functional changes in adult mouse olfactory bulb (OB) astrocytes, affecting sensory processing. Indeed, astrocyte-specific deletion of *Sox9* in the OB results in decreased odor detection thresholds and discrimination and it is associated with aberrant neuronal sensory response maps. At functional level, loss of astrocytic *Sox9* impairs the electrophysiological properties of mitral and tufted neurons. RNA-sequencing analysis reveals widespread changes in the gene expression profiles of OB astrocytes. In particular, we observe reduced GLT-1 expression and consequential alterations in glutamate transport. Our findings reveal that astrocytes are required for physiological sensory processing and we identify astrocytic *Sox9* as an essential transcriptional regulator of mature astrocyte function in the mouse OB.

## Abstract (web summary)

Astrocytes can regulate neuronal activity. Here, the authors show that astrocyte-specific deletion of Sox9 results in impaired neuronal sensory processing in the mouse adult olfactory bulb.

## Introduction

Astrocytes execute an array of roles that are essential for normal brain function^1^. Classically, astrocytes have been considered primarily a support cell, by providing metabolic balance to neurons^2^, potassium buffering^3–6^, neurovascular coupling^7,8^, glutamate recycling^9,10^, and regulating extracellular osmotic space^11^. More recently astrocytes have been recognized as active participants in synaptic neurotransmission^12,13^. Synaptic complexes that comprise astrocytic perisynaptic processes, neuronal presynaptic boutons, and postsynaptic dendritic spines form “tripartite” synapses, in which all three elements collectively operate to regulate synaptic signaling^14–17^. Nevertheless, despite their essential contributions to neuronal circuitry, specialized roles for astrocytes in region-specific circuits have only recently been appreciated, and is an emerging feature of astrocyte biology that remains relatively nascent^18–20^.

The rodent olfactory system exhibits well-defined anatomy, with astrocytes abundantly localizing at synapse-rich areas of the olfactory bulb (OB) where sensory processing initially occurs. As such, the mouse OB is a good model for investigating astrocyte-neuron interactions. During olfaction, volatile odorant molecules bind to receptors on olfactory sensory neurons (OSNs) that project to distinct glomeruli in the OB^21^, each of which are innervated by OSNs that express common olfactory receptors^22–26^. Within glomeruli, OSNs synapse with subsets of sister mitral and tufted cells (M/Ts), where OSN stimulation results in the activation of spatially-defined populations of M/T(s) that can be visualized as discrete odor response sensory maps^27–29^. This representation of external stimuli has typically been studied with attention towards the neuronal constituents of olfactory circuitry^30–32^. Interestingly, OSN activity at OB glomeruli additionally leads to activity of glomerular astrocytes and subsequent functional hyperemia^8^. Despite this known input-output relationship of glomerular astrocytes to OSN activity, the molecular and transcriptional mechanisms that underlie astrocytic contributions to olfactory circuits remain elusive.

Here we sought to investigate roles for mature astrocytes in the adult mouse olfactory system. Single-cell sequencing revealed that the transcription factor *Sox9* is selectively expressed in astrocyte populations in the OB. While *Sox9* is an established regulator of glial development^33,34^, its role in mature astrocytes remains undefined. Therefore we examined the role of *Sox9* in adult OB astrocytes, finding that its deletion drastically altered astrocycte morphology and calcium handling activity. These in vivo manipulations of astrocytes affected olfactory behaviors in mice, influencing both odor detection thresholds and odor discrimination. These observed alterations were accompanied by changes to neuronal sensory response maps, electrophysiological properties of M/T cells, and increased OSN innervation. Finally, the gene expression profiles of *Sox9*-deficient astrocytes were directly affected, highlighted by a reduction in GLT-1 and reduced glutamate transporter currents. Knockdown of GLT-1 in the OB via shRNAi generated phenotypes similar to those observed in *Sox9* KO animals, including altered astrocycte morphology and decreased odor discrimination. Together, these studies illustrate the critical contributions of astrocytes to olfactory information processing, and further define a role for *Sox9* in adult astrocytes of the OB.

## Results

**Fig. 1:**
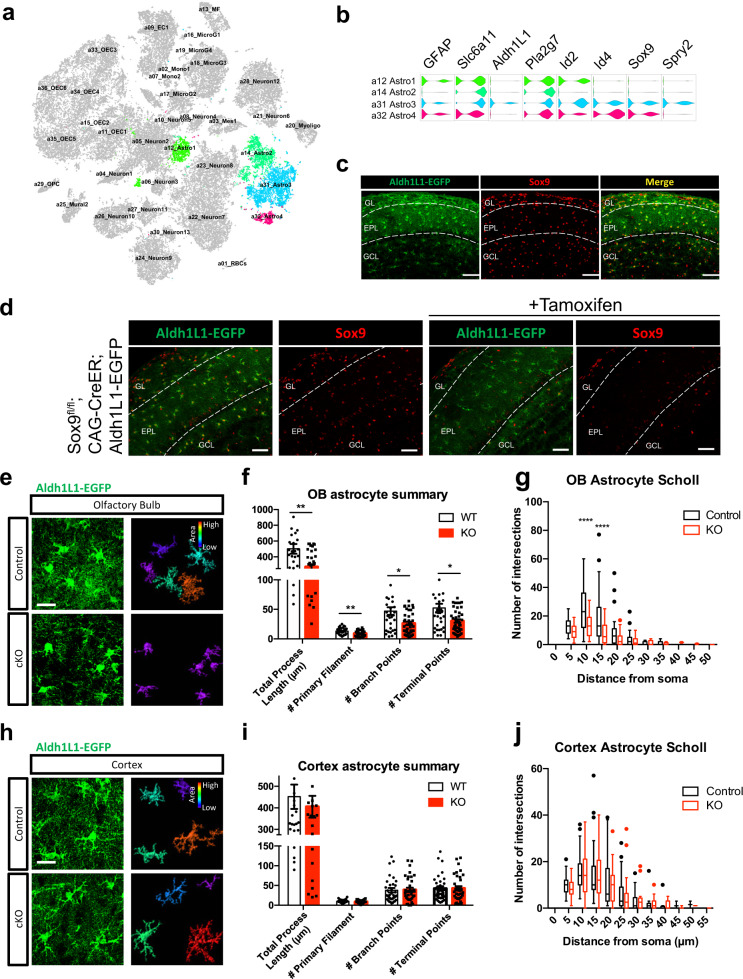
RNA-seq identified Sox9 as an important transcription factor in mature, adult astrocytes. **a** Cellular composition of the OB was visualized using t-distributed stochastic neighbor embedding (t-SNE). Single-cell sequencing allowed unbiased clustering of cell populations in the OB, revealing 36 distinct populations. Astrocyte clusters are highlighted for identification. **b** Of the 36 populations, 4 astrocyte populations were found based on expression of known astrocyte markers (Aldh1L1 and GFAP). Violin plots show common astrocyte markers along with 4 transcription factors found to be highly expressed across astrocyte populations. **c** Representative images from coronal sections from the Aldh1L1-EGFP BAC transgenic mouse line showed that immunohistochemical staining of *Sox9* colocalizes with astrocytes in the OB. *N* = 3 mice. Scale bar, 50 µm. **d** Representative images from coronal sections from the OB showing *Sox9* expression in control animals (left) and tamoxifen-induced KO of *Sox9* (right). *N* = 3 mice. Scale bar, 50 µm. **e**–**g** High-resolution imaging and surface rendering of astrocytes in OB shows a decrease in morphological branching in *Sox9* KO vs controls. *n* = 33 cells (control) and *n* = 40 cells (*Sox9* KO) from *N* = 3 mice, respectively. Adjusted *p* = 0.0021, 0.0046, 0.0219, and 0.0230 for total process length, # primary filament, # branch points, and # terminal points, respectively; 2-way ANOVA with Sidak’s multiple comparison correction. Data are presented as box plots displaying interquartile range and medians displaying interquartile range and median with Tukey whiskers. Scale bar, 20 µm. **h**–**j** High-resolution imaging and surface rendering of astrocytes in cortex shows no significant change in morphological branching in *Sox9* KO vs controls. *n* = 39 cells (control) and *n* = 35 cells (*Sox9* KO) from *N* = 3 mice, respectively. 2-way ANOVA with Sidak’s multiple comparison correction. Control in E-J is *Sox9*^fl/fl^; Aldh1L1-EGFP. Data are presented as box plots displaying interquartile range and median with Tukey whiskers. Scale bar, 20 µm.

Previously, our lab performed single-cell sequencing (Drop-seq) to determine the transcriptional profiles of different populations of cells within the adult mouse olfactory bulb^35^. To screen for prospective regulators of adult astrocyte functions, we performed unbiased clustering based on unsupervised principal-component analysis and visualization using t-distributed stochastic neighbor embedding (t-SNE) and identified 4 distinct astrocyte clusters from a total of 36 populations (Fig. 1a, S1a). We examined the first 500 highly enriched genes that showed robust expression profiles, and subsequently filtered for candidate transcription factors. Setting a threshold for 2-fold increased expression over other cell populations, filtering criteria revealed four TFs: *Id2*, *Id4*, *Sox9*, and *Spry2*. While *Id2* and *Spry2* were expressed in multiple cell type clusters, *Id4* and *Sox9* were only detected in two shared clusters, both within astrocytes and coexpressed with Aldh1L1 (Fig. 1b), a pan-astrocyte marker^36^. Immunohistochemical staining in *Aldh1L1-EGFP* BAC transgenic mice, an established mouse line that labels astrocytes with EGFP^37^, showed that expression of *Sox9* in adult animals colocalized exclusively with EGFP in the OB, indicating that its expression is restricted to astrocytes (Fig. 1c). These observations implicate *Sox9* as a key transcriptional regulator of mature astrocyte function, and thus prompted us to focus our studies on roles for *Sox9* in mature astrocytes.

Given the important role of *Sox9* during glial development and astrocyte precursors^34^, we sought to determine whether it played a separate and/or additional roles in adult OB astrocytes. Towards this, we generated mice that harbored a conditional floxed *Sox9* allele and a tamoxifen-inducible Cre driven by the CAG promoter (CAG-CreER). Due to the restricted expression of *Sox9* in astrocytes of adult brain tissue^38^, this mouse line enabled selective deletion of *Sox9* in adult astrocytes. For conditional mutagenesis, adult mice were treated with tamoxifen twice daily (150 mg/kg each) for a total of 10 days to induce genetic recombination of LoxP sites flanking *Sox9* exons 2-3^39^. Analysis of the OB 6 weeks after tamoxifen treatment confirmed efficient deletion of *Sox9* in OB astrocytes, as well as degradation and loss of existing Sox9 protein (Fig. 1d, S2a). Notably, *Sox9* deletion did not result in a reduction in the overall number of astrocytes (Fig. S2b). Given that astrocyte morphology is linked to their functions related to synaptic modulation, we sought to assess their morphological features after *Sox9* deletion. To visualize astrocyte morphology, we crossed *Sox9* conditional KO mice with Aldh1L1-EGFP BAC transgenic mice, creating a three-allele conditional reporter model (*Sox9* cKO: *Sox9*^*fl/fl*^*; CAG-CreER; Aldh1L1-EGFP* and control*: Sox9*^*fl/fl*^*; Aldh1L1-EGFP*). Upon tamoxifen-induced KO of *Sox9*, we performed multi-plane confocal imaging for surface rendering and reconstruction of OB astrocyte morphology (Fig. 1e) and observed a reduction in the number of primary filaments (13.121 ± 0.976 in WT vs 9.1 ± 0.724 in KO), branch points (47.000 ± 6.585 in WT vs 26.200 ± 2.911 in KO), terminal points (52.242 ± 7.073 in WT vs 30.275 ± 3.080 in KO), and total process length (500.791 ± 61.890 µm in WT vs 273.148 ± 27.171 µm in KO) (Fig. 1f). Moreover, Sholl analysis revealed a decrease in morphological complexity (Fig. 1g). In contrast, astrocyte morphology in the cortex was largely unaffected (number of primary filaments: 9.879 ± 0.616 in WT vs 8.486 ± 0.668 in KO, branch points: 37.949 ± 5.579 in WT vs 39.057 ± 5.431 in KO, terminal points: 42.385 ± 5.903 in WT vs 42.771 ± 5.589 in KO, and total process length: 451.685 ± 56.622 µm in WT vs 405.389 ± 50.626 µm in KO) (Fig. 1h–j). The differences we detected with *Sox9* KO between astrocyte morphologies in the OB versus the cortex suggest region-specific transcriptional dependencies on *Sox9*. These data indicate that *Sox9* is essential in the homeostatic maintenance of astrocyte morphology in the adult OB.

**Fig. 2:**
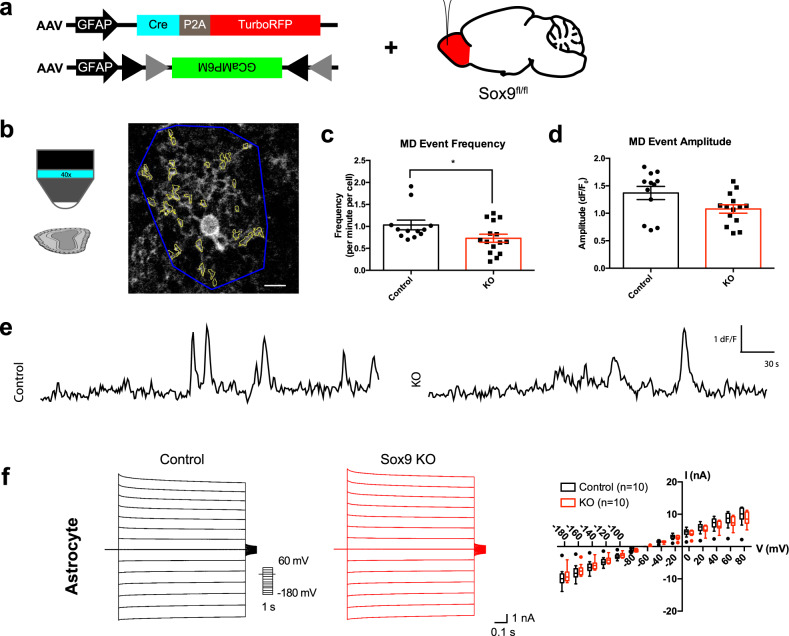
Sox9 deletion disrupted cell intrinsic properties in astrocytes. **a** Schematic of virally-induced *Sox9* KO approach and targeted expression of GCaMP in astrocytes with *Sox9* deleted. **b** Schematic of two-photon imaging of GCaMP6m from an ex vivo slice from *Sox9*^fl/fl^ animals injected with a GFAP-driven Cre virus. Images were captured at ~1 Hz over 5 min. Approximate astrocyte territory (outlined in blue) defined the range for a single astrocyte and contained multiple astrocyte microdomains (outlined in yellow). Scale bar, 10 µm. **c**, **d** Quantification of calcium events in microdomains (MDs) of astrocyte processes. Astrocytes with *Sox9* KO have a lower frequency of calcium events in microdomains but no change in amplitude. *p = 0.0435 Unpaired two-sided t-test. *n* = 12 cells from *N* = 3 control mice and *n* = 15 cells from *N* = 4 *Sox9* KO mice. Data are presented as mean values ± SEM. **e** Example traces of GCaMP6m signal in microdomains of astrocytes in control and *Sox9* KO astrocytes. **f** Whole-cell patch clamp electrophysiology of astrocytes show no changes in the I-V curve of astrocytes with stepped current injections in *Sox9* KO vs controls. *n* = 10 cells (control) and *n* = 10 cells (*Sox9* KO) from *N* = 3 mice, respectively. Data are presented as box plots displaying interquartile range and median with Tukey whiskers. Controls are C57BL/6 J injected with AAV-GFAP-iCre-P2a-TurboRFP + AAV-GFAP-flex-GCaMP6m.

We sought to further examine *Sox9* function in astrocytes from the OB. Astrocyte calcium activity is considered a key component that mediates their interactions with neurons^40–42^. To evaluate the calcium dynamics in OB astrocytes, we co-injected a viral mixture of an AAV with the astrocyte-specific GFAP promoter driving Cre expression (AAV-GFAP-iCre-P2A-TurboRFP) and a conditional AAV-GFAP-flex-GCaMP6m into the OB of *Sox9*^fl/fl^ mice to drive genetic deletion of *Sox9* as well as targeted expression of GCaMP6m (Fig. 2a). Similar injections into C57BL/6 J mice (WT) served as controls. Injection in the OB resulted in high efficiency KO of *Sox9* (39.14 ± 5.381% Sox9+; Aldh1L1-EGFP+ astrocytes in *Sox9*^fl/fl^; Aldh1L1-EGFP animals vs 86.51 ± 4.67% in Aldh1L1-EGFP controls) (Fig. S2c–e) as well as comparable changes in morphology seen through tamoxifen-induced KO (Fig. S3a–d). We harvested OB slices 4 weeks after injections and imaged calcium transients via two-photon microscopy (Fig. 2b (left), E) in both controls and *Sox9*^*fl/fl*^ mice (*n* = 3 and 4 mice of each genotype respectively). To analyze calcium events for all cells within the fields of view, we used the previously reported GECIquant software^43^ to generate semi-automated detection of regions of interest (ROIs) (Fig. 2b, right). While astrocyte somas showed no changes in calcium events, we found that *Sox9*-deleted astrocytes displayed a significant decrease in frequency of calcium events normalized specifically across the regions of microdomains (1.056 ± 0.128 events per minute in KO vs 0.730 ± 0.092 events per minute in control), and no significant change in event amplitude (Fig. 2c–e). The reduction in astrocyte microdomain calcium events are congruent with the observed changes in astrocyte morphology. Together, the defects in gross morphology and changes in astrocyte microdomain calcium activity validated functional aberrations in the cellular properties of *Sox9* KO astrocytes.

To measure whether *Sox9* KO affected biophysical properties in astrocytes, we next performed electrophysiological whole-cell patch clamp recordings. For this we injected *Sox9*^fl/fl^ animals or C57BL/6 J controls with AAV-GFAP-iCre-P2a-TurboRFP and, allowing 4 weeks for viral expression, recombination, and degradation of *Sox9* protein. Stepped current injections revealed no differences in the current-voltage relationship between *Sox9* KO and control cells, displaying characteristic linear IV curve with similar slopes (Fig. 2f), suggesting that deletion of *Sox9* in astrocytes did not affect their general membrane properties.

**Fig. 3:**
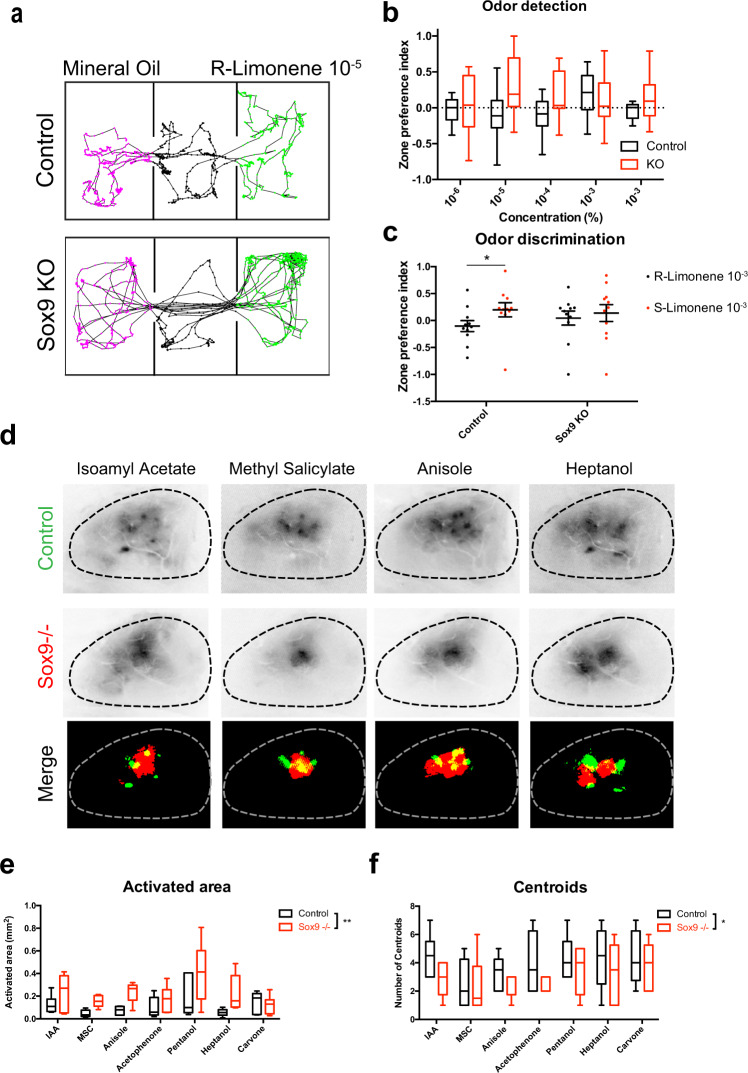
Deletion of the astrocyte specific marker Sox9 dysregulated olfactory behaviors and sensory maps. **a** Schematics of live mouse tracking during three compartment place preference assay. Top represents a control mouse investigating the both chambers equally with the odor present at 10^−5^. Bottom represents a *Sox9* KO mouse preferentially investigating the odor chamber at 10^−5^. **b** Quantification of (**a**). *n* = 11 (*Sox9* KO) and *n* = 11 (Control). Two-way RM ANOVA with Bonferroni’s multiple comparison test (main effect of group, F(1,20) = 1.913, *p* = 0.1819; group X concentration interaction, F(3,60) = 3.385, *p* = 0.0238). Data are presented as box plots displaying interquartile range and median with Tukey whiskers. **c** Whisker plot quantifying three chamber place preference during odor discrimination. *n* = 11 (*Sox9* KO) and *n* = 11 (Control). Two-way RM ANOVA with Bonferroni’s multiple comparison test (main effect of odor, F(1,20) = 5.895, *p* = 0.0247; group X odor interaction, F(1,20) = 1.660, *p* = 0.2123). Data are shown as mean ± s.e.m. **d** Representative images of neuronal olfactory sensory maps from 4 different odorants of control animals vs astrocyte *Sox9* KO animals. Merged image shows changes in olfactory sensory maps after binary thresholding. **e**, **f** Quantification of change in activated area (main effect of group, F(1,67) = 9.056, *p* = 0.0037) and number of centroids (main effect of group, F(1,70) = 6.235, *p* = 0.0149) in *Sox9* KO animals vs controls. *n* = 6 mice per group. Two-way ANOVA with Sidak’s multiple comparison correction. Data are presented as box plots displaying interquartile range and median with Tukey whiskers. Control in A-C is C57BL/6 J injected with AAV-GFAP-iCre-P2a-TurboRFP; control in **d**–**f** is Thy1-GCaMP3 injected with AAV-GFAP-iCre-P2a-TurboRFP.

Calcium activity in astrocytes is considered an indirect measure of astrocyte-neuron interactions, that can ultimately influence neuronal activity^44,45^. Therefore, the observed defects in calcium activity in astrocytes lacking Sox9 raised the question of whether astrocyte-neuron signaling is altered by deletion of *Sox9* in OB astrocytes. To address this, we first assayed animals for potential alterations in olfactory related behaviors by injecting AAV-GFAP-iCre-P2a-TurboRFP into *Sox9*^*fl/fl*^ mice to selectively knock-out *Sox9* in OB astrocytes, or into C57BL/6 J mice as controls. Four weeks after injection, allowing ample time for viral expression, recombination, and degradation of Sox9 protein, we performed odor detection and olfactory habituation/dishabituation assays using a 3-compartment place preference assay (3PP). Mice were habituated to a 3-compartment chamber where the outer compartments contained mineral oil and the middle compartment served as a neutral barrier. After habituation, we swapped mineral oil with a novel odorant ((R)-Limonene) in one compartment in a four-step ascending concentration series and measured the time that mice spent in that compartment investigating the odor versus mineral oil. Control mice investigated both mineral oil and (R)-Limonene compartments equally until odor concentrations of 10^−3^ (v/v) of (R)-Limonene, in which control mice then spent more time in the compartment with the odor (Zone preference index = 0.198 ± 0.096)(Fig. 3a, b), suggesting novel odor detection. Somewhat surprisingly, *Sox9* KO mice spent more time investigating odorants at concentrations at dilutions to 10^−5^ (v/v) (Zone preference index = 0.279 ± 0.122). Similar detection results were obtained when (+)-Carvone was tested (Fig. S4a) with *Sox9* KO mice trending towards increased odor sensitivity compared to controls. With isoamyl acetate, a non-preferred odor, mice spent more time investigating the compartment with mineral oil upon odor detection but showed no significant changes in odor sensitivity at the concentrations tested in *Sox9* KO mice as compared to controls (Fig. S4b). Together, these data suggest that mice with *Sox9* deleted from olfactory bulb astrocytes exhibit decreased olfactory detection thresholds and thus increased odor sensitivity.

Next, to assay for detection of differences in olfactory discrimination^46^, we used a related paradigm to examine fine odor discrimination by introducing the novel, but structurally similar odorant (S)-Limonene. Upon habituating mice to (R)-Limonene (10^−3^), we then introduced (S)-Limonene (10^−3^). In the presence of (S)-Limonene, control mice spent significantly more time in the compartment investigating the novel odor (Zone preference index = −0.104 ± 0.102 for (R)-Limonene vs 0.199 ± 0.131 for (S)-Limonene), whereas *Sox9* KO mice showed no significant differences in compartment preference (Zone preference index = 0.045 ± 0.130 for (R)-Limonene vs 0.138 ± 0.157 for (S)-Limonene) (Fig. 3c). Together, these data suggest that *Sox9* KO mice have more sensitive odor detection thresholds, but are deficient in their ability to discriminate between chemically similar but perceivably different odors. These observations indicate that Sox9 plays a critical role in astrocyte regulation of circuit-level sensory processing and the behavioral outcomes associated with olfactory circuit function.

The observed aberrant olfactory behavioral phenotypes next led us to investigate whether olfactory bulb neurons and their circuits are impacted non-cell-autonomously by *Sox9* KO in OB astrocytes. We first examined whether *Sox9* deletion in OB astrocytes altered olfactory sensory response maps. Towards this, we generated *Sox9*^*fl/fl*^*;* Thy1-GCaMP3 mice, that expressed the genetically-encoded calcium indicator GCaMP3 in M/T cells, and used the viral-based approaches described above to selectively knock-out (KO) *Sox9* in OB astrocytes while monitoring odor-evoked calcium responses in M/T cells. After injecting AAV-GFAP-iCre-P2a-TurboRFP into *Sox9*^fl/fl^; Thy1-GCaMP3 mice to selectively KO *Sox9* in OB astrocytes or Thy1-GCaMP3 mice^47^ as controls, we imaged the dorsal surface of the olfactory bulb for potential changes in sensory response map properties to a panel of different odorants (Fig. 3d). Notably, we found aberrant changes to the area of activation of the imaged neuronal odor sensory maps in the *Sox9*-KO OB compared to controls (Fig. 3e, f). By implementing automated maximum entropy thresholding approaches, we observed that the area activated by odorants significantly increased (Fig. 3e), along with a decrease in the number of centroids (Fig. 3f) in experimental animals compared to controls, suggesting altered activated domains and OB sensory maps. Given the changes in sensory response maps of M/T cells in *Sox9* KO mice, we sought to determine if glomerular olfactory maps were altered by loss of *Sox9*. To observe glomerular olfactory maps, we examined OSN inputs by staining for olfactory marker protein (OMP), a key marker of mature OSNs important in regulating formation and refinement of glomeruli^48^. In coronal sections of the OB, we observed an increase in the OMP density per glomerulus (37,821 ± 3,155 µm^3^/glomerulus in control mice vs 59,621 ± 4,384 µm^3^/glomerulus in *Sox9* KO mice), as well as increased glomerular size (37,821 ± 3,155 µm^3^ in control mice vs 59,621 ± 4,384 µm^3^ in *Sox9* KO mice) in *Sox9* KO mice (Fig. S4c–h), suggesting increased OSN innervation into glomeruli after *Sox9* deletion in OB astrocytes. Together, these data implicate Sox9 as an important molecular player in astrocytes in regulating neuronal sensory maps in the OB and support the notion that neuron-astrocyte coordination is disrupted in the absence of Sox9.

**Fig. 4:**
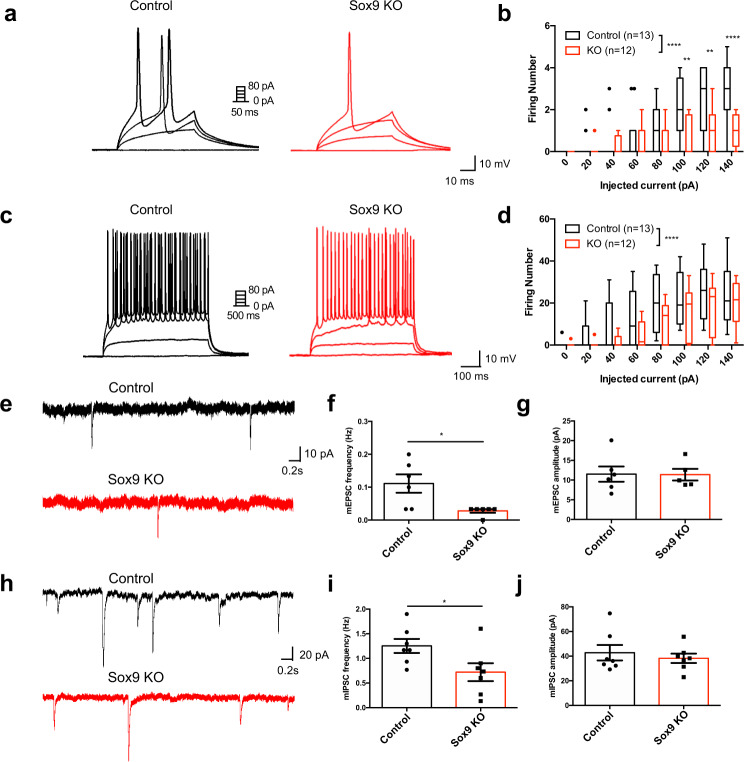
Electrophysiological recordings showed a reduction in evoked APs in M/T as well as altered synaptic properties. (**a**–**d**) Whole-cell patch clamp electrophysiology of M/T cells revealed a reduction in firing number from stepped current injections with *Sox9* KO in astrocytes vs control astrocytes. *n* = 13 cells (control) and *n* = 12 cells (*Sox9* KO) from *N* = 3 mice, respectively. **p = 0.0023 and 0.0014 for 100 and 120 pA current injections, respectively; ****p < 0.0001; Two-way ANOVA with Sidak’s multiple comparison correction. Data are presented as box plots displaying interquartile range and median with Tukey whiskers. **e** Example traces of mEPSC recordings in M/T cells with control astrocytes (top) and *Sox9* KO astrocytes (bottom). **f** M/T cells with *Sox9* KO astrocytes show a significant decrease in mEPSC frequency compared to control astrocytes. *n* = 8 cells (control) and *n* = 7 cells (*Sox9* KO) from *N* = 3 mice, respectively. *p = 0.0156. Two-tailed Student’s t-test. Data are presented as mean values ± SEM. **g** M/T cells show no changes in mEPSC amplitude with *Sox9* KO in astrocytes. *n* = 8 cells (control) and *n* = 7 cells (*Sox9* KO) from *N* = 3 mice, respectively. Two-tailed Student’s t-test. **h** Example traces of mIPSC recordings in M/T cells with control astrocytes (top) and *Sox9* KO astrocytes (bottom). Data are presented as mean values ± SEM. **i** M/T cells with *Sox9* KO astrocytes show a significant decrease in mIPSC frequency compared to control astrocytes. *n* = 7 cells (control) and *n* = 7 cells (*Sox9* KO) from *N* = 3 mice, respectively. *p = 0.0392. Two-tailed Student’s t-test. Data are presented as mean values ± SEM. **j** M/T cells show no changes in mIPSC amplitude with *Sox9* KO in astrocytes. *n* = 7 cells (control) and *n* = 7 cells (*Sox9* KO) from *N* = 3 mice, respectively. Two-tailed Student’s t-test. Control is C57BL/6 J injected with AAV-GFAP-iCre-P2a-TurboRFP. Data are presented as mean values ± SEM.

The previous data illustrate both altered astrocyte function and astrocyte-neuron communication, which led us to evaluate whether electrophysiological properties of neurons were also affected when *Sox9* was deleted from astrocytes. Towards this, we injected AAV-GFAP-iCre-P2a-TurboRFP into the OB of *Sox9*^fl/fl^ mice to selectively knock out *Sox9* in OB astrocytes, or into C57BL/6 J mice as controls. We performed whole-cell recordings from M/T cells since these cells directly receive odor information from olfactory sensory neurons and serve as the primary neuronal output from the OB to higher order brain regions involved in odor processing. To determine if other active response properties were affected, we applied stepped current injections at 20 pA increments. In animals with *Sox9* deletion, M/T cells showed a significant decrease in the number of evoked action potentials during both short (50 ms, Fig. 4a, b) and long (500 ms, Fig. 4c, d) pulses compared to controls. These data reveal that M/T cell function is altered in the presence of *Sox9*-KO astrocytes, indicating that the alterations in astrocyte properties driven by *Sox9* have direct effects on neuronal substrates within the OB. Given that M/T cell physiological properties were affected, and that astrocytes contribute to synaptic function, we next examined if synaptic properties were affected. Notably, while performing targeted whole cell recordings in M/T cells we found that mEPSC and mIPSC frequencies were reduced (mEPSC: 0.1111 ± 0.0281 Hz in controls vs 0.0277 ± 0.0055 Hz in *Sox9* KO; mIPSC: 1.252 ± 0.1425 Hz in controls vs 0.7190 ± 0.1811 Hz in *Sox9* KO) with no observable changes in amplitudes (Fig. 4e–j), indicating fewer synaptic connections following *Sox9* KO.

Detecting altered synaptic properties in M/T cells associated with Sox9-deficient astrocytes, we next queried the specificity of this effect by examining whether other neuronal subtypes in the OB were affected. Granule cells are the most prevalent interneuron cell-type in the OB, and are known to make dendrodendritic connections with M/T cells^49,50^. We observed no significant changes in the electrophysiological properties of granule cells (Fig. S5a, b), indicating that *Sox9* deletion affected neuron-astrocyte interactions in a cell-type specific manner within the OB, with pronounced effects manifesting in M/T cells.

**Fig. 5:**
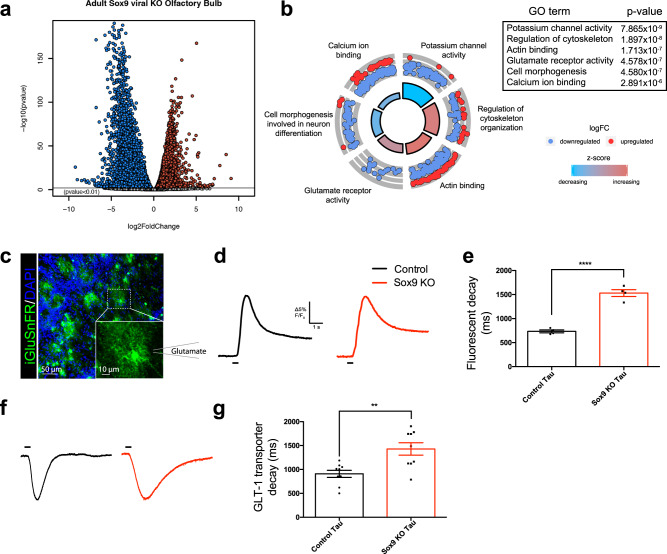
RNAseq revealed a significant change in transcription profile of adult, Sox9-deleted astrocytes. **a** Volcano plot of genes significantly upregulated (red dots) and downregulated (blue dots) in *Sox9*-deleted astrocytes compared to controls. **b** Downregulated genes from *Sox9* deletion revealed GO terms associated with neurotransmitter receptor and ion channel activity, as well as cellular morphology. **c** Representative high resolution image of iGluSnFR expressed in astrocytes in the OB. *N* = 4 mice. Inset: high magnification of a single astrocyte with schematic of glutamate puffer nearby astrocyte. **d** Example trace of iGluSnFR signal from control and *Sox9* KO astrocytes after puffing glutamate [534 mM]. Black line indicates duration of glutamate puff. **e** Quantification of (**d**). *Sox9* KO astrocytes had a significantly increased decay after puffing glutamate. *n* = 37 cells (control) and *n* = 35 cells (*Sox9* KO) from *N* = 4 mice, respectively. ****p < 0.0001. Two-tailed Student’s t-test. Data are presented as mean values ± SEM. **f** Example peak normalized traces from whole cell electrophysiological recordings of glutamate transporter currents in astrocytes. Black line indicates duration of glutamate puff. **g** Quantification of (**f**). *Sox9* KO astrocytes had a significantly increased decay after puffing glutamate. *n* = 9 cells (control) and *n* = 9 cells (*Sox9* KO) cells from *N* = 4 and 3 mice, respectively. **p = 0.0032. Two-tailed Student’s t-test. Controls in A-B and F-G are C57BL/6 J injected with AAV-GFAP-iCre-P2a-TurboRFP; controls in C-E are C57BL/6 J injected with AAV-GFAP-iGluSnFR. Data are presented as mean values ± SEM.

The observed changes in astrocyte morphology and calcium activity (Figs. 1 and 2), coupled with Sox9’s role as a potent transcription factor, suggest that Sox9-deficient astrocytes exhibit widespread changes in gene expression. To assay for such changes in gene expression, and assess the impact on astrocyte-neuron interactions, we next performed whole-transcriptome RNA-seq expression analysis on fluorescence-activated cell sorting (FACS)-isolated *Sox9* KO astrocytes from the OB^51^. AAV-GFAP-iCre-P2a-TurboRFP was injected into *Sox9*^fl/fl^ mice or C57BL/6 J control mice. After 4 weeks to allow for deletion of *Sox9* DNA and degradation of *Sox9* mRNA and protein, astrocytes were FACS-isolated using TurboRFP as a marker for astrocytes. RNA-seq expression analysis revealed pronounced dysregulation of gene expression; using *p* < 0.05 and a threshold of 1.5-fold change in expression, our analysis identified over 2,500 differentially expressed genes (407 upregulated in KO and 2,057 downregulated in KO) (Fig. 5a). From the list of significantly upregulated or downregulated genes, further analysis of the expression profile of *Sox9* deleted astrocytes with gene ontology (GO) revealed a significant reduction in expression in genes related to channel activity, ion balance, calcium signaling, and importantly, the response to neuronal circuit activity (Fig. 5b; Fig. S6c). This analysis revealed that *Sox9*-deleted astrocytes significantly downregulated genes associated with physiological processes essential for neuronal function, astrocyte cellular structure, and astrocyte morphology. Importantly, the observed changes in gene expression were congruent with phenotypic analysis of *Sox9*-deficient astrocytes, as they exhibited notable changes in cellular morphology (Fig. 1e–g) and suppression of calcium activity (Fig. 2b–d). Alterations in astrocyte form and function subsequently exert themselves onto neurons manifesting in the OB as M/T cell activity disruption, further suggesting that *Sox9* KO may exhibit defects in neuron-astrocyte coordination and/or communication in the OB. qPCR verification of select target genes encoding astrocyte receptors and channels responsible for neuronal synaptic responses and homeostatic scaling mechanisms confirmed the changes in gene expression revealed by RNA-seq (Fig. S6d).

Further analysis of the RNA-seq data revealed downregulation of genes such as Syt11, which is a known factor that regulates membrane trafficking and exocytosis^52–54^, and Slc1a2 (GLT-1), the predominant glutamate transporter in astrocytes that participates in reuptake of glutamate from the synaptic cleft. Given these transcriptional changes, the observed changes in sensory responses, and altered M/T cell synaptic function, we next queried if these phenotypes may be linked to altered astrocytic glutamate handling. Towards this, we employed the glutamate-sensing reporter iGluSnFR^55^ to assay for potential changes in *Sox9* KO astrocyte glutamate uptake from the reduction of GLT-1 RNA. We injected AAV-GFAP-iCre-P2a-TurboRFP with AAV-GFAP-iGluSnFR into *Sox9*^fl/fl^ animals to selectively express iGluSnFR in *Sox9* KO astrocytes, or AAV-GFAP-iGluSnFR alone into C57BL/6 J animals as controls. With iGluSnFR expressed in *Sox9* KO astrocytes or controls, respectively, we performed widefield ex vivo imaging with puff administration of glutamate (500 mM in ACSF) to observe responses to extracellular glutamate (Fig. 5c). Each puff of glutamate evoked robust and short-lived iGluSnFR fluorescent signals (Fig. 5d). We assessed the rate of glutamate uptake by measuring the rate of decay of iGluSnFR signal, and observed that the signal decay was far slower in *Sox9* KO astrocytes compared to that observed in control astrocytes (Fig. 5e), suggesting altered and slower glutamate uptake with loss of Sox9.

To independently confirm the results of the iGluSnFR optical sensor, we next performed targeted electrophysiological studies. Using whole-cell patch clamp recordings from control and *Sox9* KO astrocytes, we applied local puffs of glutamate (500 mM in ACSF) adjacent to recorded astrocytes. We recorded baseline glutamate mediated currents, followed by currents after application of TBOA to block glutamate transporters (Fig. 5f), and observed that indeed the decay constant of the subtracted glutamate transport was significantly longer in *Sox9* KO astrocytes compared to controls (1429 ± 130.0 ms vs 908.4 ± 74.81 ms, respectively) (Fig. 5g).

**Fig. 6:**
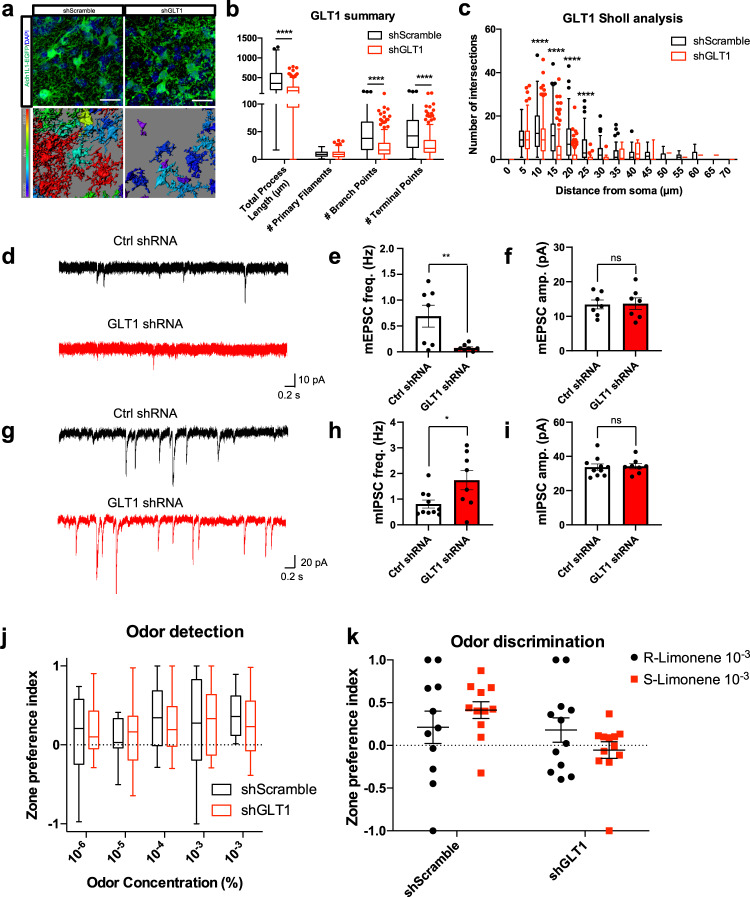
GLT-1 knockdown partially recapitulates Sox9 KO phenotypes. **a**–**c** High-resolution imaging and surface rendering of astrocytes in OB shows a decrease in morphological branching in shGLT1 vs shScramble. *n* = 256 cells (shGLT1) and *n* = 138 cells (shScramble) from *N* = 3 mice, respectively. ****p < 0.0001; 2-way ANOVA with Sidak’s multiple comparison correction. Quantification of (**a**) represented by box plots displaying interquartile range and median with Tukey whiskers. Data are presented as mean values ± SEM. Scale bar, 20 µm. **d** Example traces of mEPSC recordings in M/T cells with shScramble control injections (top) and shGLT1 KD injections (bottom). **e** M/T cells with shGLT1 injections show a significant decrease in mEPSC frequency compared to control injections. n = 7 cells (shScramble) and *n* = 7 cells (shGLT1) from *N* = 4 mice, respectively. **p = 0.0084. Two-tailed Student’s t-test. Data are presented as mean values ± SEM. **f** M/T cells show no changes in mEPSC amplitude with shGLT1 injections. *n* = 7 cells (shScramble) and *n* = 7 cells (shGLT1) from *N* = 4 mice, respectively. Two-tailed Student’s t-test. Data are presented as mean values ± SEM. **g** Example traces of mIPSC recordings in M/T cells with shScramble control injections (top) and shGLT1 KD injections (bottom). **h** M/T cells with shGLT1 injections show a significant increase in mIPSC frequency compared to control injections. *n* = 10 cells (shScramble) and *n* = 7 cells (shGLT1) from *N* = 4 mice, respectively. *p = 0.0257. Two-tailed Student’s t-test. Data are presented as mean values ± SEM. (**i**) M/T cells show no changes in mIPSC amplitude with Sox9 KO in astrocytes. *n* = 10 cells (shScramble) and n = 7 cells (shGLT1) from *N* = 4 mice, respectively. Data are presented as mean values ± SEM. **j** Quantification of 3 compartment place preference assay. *n* = 12 (shGLT1) and n = 11 (shScramble). Two-way RM ANOVA with Bonferroni’s multiple comparison test (main effect of group, F(1,21) = 0.0002768, *p* = 0.9869; group X concentration interaction, F(4,84) = 0.2148, *p* = 0.9295). Data are presented as box plots displaying interquartile range and median with Tukey whiskers. **k** Whisker plot quantifying three chamber place preference during odor discrimination. *n* = 12 (shGLT1) and *n* = 11 (shScramble). Two-way RM ANOVA with Bonferroni’s multiple comparison test (main effect of odor, F(1,21) = 0.02911, *p* = 0.8662; group X odor interaction, F(1,21) = 4.663, *p* = 0.0425). Data are shown as mean ± s.e.m.

Given that loss of *Sox9* results changes in glutamate uptake, we sought to address the contributions of GLT-1 dysregulation on the previously identified phenotypes. To determine the impact of downregulation of GLT-1 on these other phenotypes, we used viral delivered GLT-1 shRNA to selectively knockdown GLT-1 transcription^56^. After viral injection and recovery, viral delivered shGLT1 with EFi1-driven TurboRFP marker resulted in a significant reduction in GLT-1 mRNA normalized to GAPDH, and compared to shScramble-injected controls (Fig. S6e). We next injected AAV-shGLT-1 or AAV-shScramble controls into the OB of Aldh1L1-EGFP animals. After allowing 5 weeks for efficient knockdown of GLT-1 mRNA, we assayed for changes in astrocyte morphology by performing multi-plane confocal imaging and reconstruction. Similarly to *Sox9* KO astrocytes, we observed a significant reduction in the number of branch points (46.397 ± 3.161in shScramble injected animals vs 22.543 1.052 in shGLT1 injected animals), terminal points (50.463 ± 3.276 in shScramble vs 26.421 ± 1.14 in shGLT1), and total process length (419.517 ± 24.782 µm in shScramble vs 197.307 ± 8.069 µm in shGLT1) (Fig. 6a, b). Sholl analysis also revealed a significant reduction in morphological complexity (Fig. 6c).

To determine the effects of GLT-1 knockdown in the OB, we next examined for potential changes in synaptic properties. We found decrease mEPSC frequencies, but mIPSC frequencies were increased (mEPSC: 0.6905 ± 0.2117 Hz in controls vs 0.0750 ± 0.0225 Hz in shGLT1; mIPSC: 0.8100 ± 0.1568 Hz in controls vs 1.742 ± 1.068 Hz in shGLT1). With no observable changes in amplitudes (Fig. 6d–i), knock down of GLT-1 exerted differing effects on inhibitory and excitatory synapses in the OB.

With the changes in astrocyte morphology and synaptic connections in the OB following GLT-1 KD, we next sought to determine the effects of olfactory sensitivity and discrimination using the 3 compartment place preference assay as described above. In control mice injected with shScramble in the OB, mineral oil and (R)-Limonene compartments were investigated equally until odor concentrations of 10^−4^ (v/v) of (R)-Limonene, in which control mice then spent more time in the compartment with the odor (Zone preference index = 0.364 ± 0.130), suggesting novel odor detection. Mice injected with shGLT1 spent more time investigating odorants at concentrations at dilutions to 10^−3^ (v/v) (Zone preference index = 0.294 ± 0.133) (Fig. 6j). However, two-way repeated measures ANOVA revealed no significant differences by factor of group, odor, or interaction.

To assay for odor discrimination, we habituated mice to (R)-Limonene before introducing (S)-Limonene. In the presence of (S)-Limonene, control mice spent more time in the compartment investigating the novel odor (Zone preference index = 0.2119 ± 0.1887 for (R)-Limonene vs 0.4129 ± 0.09874 for (S)-Limonene), whereas GLT-1 KD mice showed no significant differences in compartment preference (Zone preference index = 0.1801 ± 0.1419 for (R)-Limonene vs −0.05538 ± 0.09721 for (S)-Limonene) (Fig. 6k). Two-way repeated measures ANOVA revealed a significant interaction between both group and odor. Together, these data suggest that GLT-1 KD mice have no change to odor detection thresholds compared to scrambled controls, but have altered ability to discriminate between chemically similar but perceivably different odors. Collectively, these findings reveal that astrocytes play an important role in sensory processing through neuron-astrocyte interactions. The specific phenotypes of altered neuronal properties and abnormal odor discrimination that are similar to *Sox9* KO mice suggests that altered glutamate handling contributes to the observed phenotypic abnormalities in *Sox9* KO mice.

## Discussion

Here we performed astrocyte-specific KO of *Sox9* in the mouse olfactory bulb, and identify roles for astrocytes in direct modulation of sensory circuits. Combining genetic and viral manipulations, electrophysiological recordings, and in vivo imaging methods, we found that Sox9 plays an important role in maintaining proper function and transcriptional maintenance of mature, adult astrocytes. Notably, we found that astrocytic modulation of neurons not only affected their activity, but also behavioral output associated with both odor detection and odor discrimination tasks. Together these data reveal a role for Sox9 in governing mature astrocyte function, and more broadly, supports diverse roles for astrocyte-neuron coordination in sensory processing system.

*Sox9* associates with the gliogenic switch that causes neural progenitors to transit from neurogenesis to gliogenesis^33,34^. Despite well-known functions for *Sox9* in embryonic glial development and the perdurance of Sox9 expression in the adult brain^38^, roles for *Sox9* in mature adult astrocytes remain broadly unstudied. The approaches utilized here to selectively investigate astrocytes (tamoxifen-inducible and viral) enabled us to bypass Sox9’s role in development and interrogate its function in the adult brain, where we found that Sox9 exerts key roles in maintaining astrocyte morphology and communication with OB neurons. In addition, GO analysis of transcriptome data revealed downregulation of many genes related to maintaining cellular morphology and governing physiological responses, both of which are key roles in the supportive functions of astrocytes. These observations, coupled with phenotypic analysis of Sox9-deficient astrocytes, illustrate that Sox9 regulates the expression of many genes that are essential for the homeostatic function of adult astrocytes, and that these functions are critical to normal neuronal output and sensory processing. Noticably, we observed varying effects on astrocyte morphology from deletion of *Sox9* between the OB and cortex, suggesting that Sox9 may play alternative roles in astrocytes across brain regions. Indeed recent studies from our group revealed that NFIA plays a selective role in maintaining astrocyte function in the adult hippocampus, but not in the OB, cortex, or brainstem^20^. Together, these findings suggest that astrocyte function is regulated by region-sepcific transcriptional codes, where astrocytes from diverse brain regions exhibit unique transcriptional dependencies.

Nevertheless, the downregulation of genes linked to key physiological and homeostatic functions of astrocytes (Fig. 5a, b) indicates that Sox9 continues to play a key role in astrocyte function through adulthood (Figs. 2–4). The consequences of such Sox9-dependent transcriptional changes extend beyond the cell autonomous functions of astrocytes, as neuronal properties within the olfactory circuit, sensory response maps, and ultimately olfactory behaviors were altered. Together, these findings provide a molecular, cellular, and physiological framework for dissecting how transcriptional mechanisms within astrocytes influence neuronal circuit processing that can likely be extended to other brain regions. Critically, how diverse cellular functions of astrocytes are regulated at the transcriptional level have remained poorly defined. Recent studies have highlighted the regional and local diversity of astrocytes^18,44,51,57^, and it will be important to decipher whether this diversity is encoded in part, by specific transcription factors.

Sensory maps are established and maintained through multiple mechanisms^58^, ranging from developmental molecular signaling cues to activity-dependent refinement underlying synaptic plasticity. Our studies reveal a potential role for astrocytes in sensory response maps within the OB. We observed that *Sox9* KO resulted in increased OSN density at glomeruli accompanied by olfactory behavior characterized by increased olfactory sensitivity and decreased discrimination. It is well established that Sox9 expression is exclusively expressed in duct/gland cells in the olfactory epithelium but not in OSNs in both rodent and human^59–61^. Increased innervation from OSNs to glomeruli after *Sox9* cKO in OB astrocytes suggests that OSNs are also under astrocyte-mediated regulation. The increased OSN innervation may contribute to the increased olfactory sensitivity because of the increased signal input^62–64^.

Glomerular structures include astrocyte cell bodies that reside along their periphery, astrocytic processes enclosing neuropil in a central-synaptic subcompartment^65^. Astrocytes of the same glomerulus form a large multicellular syncytium through gap junctions located at their perisynaptic processes^66^, which can lead to single unit functional glial network responses characterized by calcium waves that propagate through astrocyte clusters within odor-activated glomeruli^67,68^. The anatomical arrangement of astrocytes at glomeruli coupled via glial networks may serve to constrain M/T cells responses, as revealed through a decrease in current-evoked firing rate of M/T cells (Fig. 4a–d) as well as synaptic events (Fig. 4e–j). However, we also observed increased activated area of M/T cells (Fig. 3d–f) which is counterintuitive to the decreased firing rate. The decrease in inhibitory inputs (Fig. 4h–i) may explain these contrasting results, as inhibitory inputs contribute to M/T cell selectivity^69^ and are closely tied to lateral inhibition and contrast enhancement^32,50^. Overall synchrony between astrocyte anatomy, spatial localization, and range of functions may coordinate effects onto neurons at the cellular level through neuron-astrocyte interactions, which then manifests into circuit-level phenomena.

From these data, we consider a model in which central astrocyte transcriptional regulation is important in maintaining sensory circuits through expression of receptors and transporters for homeostatic mechanisms to threshold neuronal responses. Upon *Sox9* deletion, we observed altered sensory maps and behavioral phenotypes. These roles may be maintained by a combination of precise glomerular structure, synaptic stability, and the reuptake of glutamate from the synaptic cleft. With a decrease in morphological complexity and a reduction in GLT-1 expression, reuptake of glutamate is impaired. The clearance of extracellular glutamate is mostly the responsibility of glial glutamate transporters^70,71^. Knockdown of GLT-1 confirmed that some of the phenotypes seen in *Sox9* KO such as loss of morphological complexity (Figs. 1e–g and 6a–c) and excitatory synaptic stability (Figs. 4e–g and 6d–f) is, in part, due to the loss of GLT-1. The imperfect phenocopy of GLT-1 KD to *Sox9* KO phenotypes highlights the widespread effects of loss of a key transcription factor via dysregulation of its downstream targets and the concomitant compensatory mechanisms. Interestingly, GLT-1 KD also resulted in independent compensatory mechanisms as seen by an increase in inhibitory synaptic stability (Fig. 6g–i). A possible consideration is that although previous studies indicate neuronal GLT-1 KO mice have normal perfomances on a wide variety of behavior tests^72^, non-specific expression of GLT-1 shRNA and Ef1a-turboRFP might result in certain level of the metabolic stress in both astrocytes and neurons^73^, and introduce extra variations into the astrocyte-neuron coordination.

Taken together, these experiments expand our current understanding of the roles for Sox9 in mature, adult astrocytes and their functions in neuronal circuit processing; rather than a neurocentric view, sensory circuits include a functional and cooperative role for astrocytes and their neuronal constituents in establishing and regulating the integration of sensory input in a multidimensional system. Overall, our study reinforces the need to examine interactions between multiple cell types toward unraveling how these interactions contribute towards sensory circuit function. To fully understand the complexity of how the brain shapes behavior, further investigation into glia subtypes and their interactions with neurons is imperative for processing the wide array of inputs into neuronal circuits.

## Methods

### Experimental mouse model

All procedures performed on mice were carried out in accordance with the ethical guidelines of the National Institutes of Health and approved by the institutional review board (IACUC Baylor College of Medicine) under protocol #AN-5596. All mice were housed with food and water available ad libitum in a 12-h light/dark environment. Both male and female mice were used for all experiments, and mice were randomly allocated to experimental groups. For ex vivo and in vivo experiments, adult mice aged >8 weeks were used, unless otherwise described.

### Method details

#### Plasmid generation

For GLT-1 shRNAi knockdown experiments, AAV shRNA delivery plasmids were designed based on a previous study^20^. In summary, AAV shRNA delivery backbone was acquired from Addgene (Addgene #85741)^74^. For GLT-1 RNAi, the dsDNA fragment containing target sequence against mouse GLT-1 (5′- GCTCTCACTGACTGTGTTT-3′)^56^ and the EFi1 promoter sequence followed by TurboRFP coding region were synthesized and cloned into BamHI and EcoRV sites of parental backbone next to the U6 promoter, resulting in the shGLT1 construct. For the Scramble control, original scramble sequence (5′-GTTCAGATGTGCGGCGAGT-3′) under the U6 promoter was retained, but the EF1a promoter sequence followed by turboRFP coding region were cloned into SalI and EcoRV sites of parental backbone.

#### Stereotaxic microinjection of adeno-associated viruses

Surgical procedures were conducted under general anesthesia using Ketamine/dexdormitor (75 mg/kg and 0.5 mg/kg respectively) induction and maintenance under volatile isoflurane inhalation (maintenance at 1–2.5% v/v). Depth of anesthesia was monitored continuously and adjusted when necessary. Following induction of anesthesia, the mice were fitted into a stereotaxic frame with their heads secured by blunt ear bars and their noses placed into an anesthesia and ventilation system. Mice were administered 5 mg/kg of meloxicam subcutaneously prior to surgery. The surgical site was then cleaned three times with 10% iodine and 70% isopropanol (v/v). Skin incisions were made, followed by craniotomies of 2–3 mm in diameter above the olfactory bulb using a small steel burr (Hager & Meisinger GmbH) powered by a high-speed drill (Vector Mega Torque). Bilateral viral injections in the olfactory bulb were carried out by using a stereotaxic apparatus (Leica Biosystems) to guide the placement (from bregma: ML, ±0.9 mm; AP, 3.82 mm; and 0.65 mm down from the surface of the OB) of beveled glass pipettes (3-000-203-G/X, Drummond Scientific Company). Adeno-associated viruses (AAVs) were injected by using a syringe pump (Nanoject II, Drummond Scientific Company) at a rate of 69 nL/s at 25 s intervals 10 times to obtain uniform labeling of astrocytes. Glass pipettes were left in place for at least 5 min prior to slow withdraw. Surgical wounds were closed with surgical nylon monofilament sutures. Mice were allowed to recover overnight in cages placed partially on a low-voltage heating pad. Meloxicam was administered once a day for 3 days after surgery. Virus injected mice were euthanized two to three weeks post-surgery for in vivo widefield imaging, live slice imaging, or perfused for immunohistochemistry. AAVs encoding flexed-GCaMP6m, iCre-P2a-TurboRFP, or iGluSnFR under the GFAP promoter (serotype 2/9 or DJ8, ~2.5 × 10^12^ genome copies/mL), shScramble or shGLT1 (serotype 2/9, ~1.5 × 10^12^ genome copies/mL) were cloned and packaged in house.

#### Tamoxifen treatment

For the experiments, Tamoxifen (Sigma, T5648) was dissolved in corn oil (Sigma, C8267) at 30 mg/ml. At 4 weeks old, 150 mg/kg Tamoxifen was given to both control and cKO mice by oral gavage twice a day for 5 days. Mice were allowed to recover for a week, and then 150 mg/kg Tamoxifen were given twice a day for another 5 days.

#### Immunohistochemistry and confocal imaging

For confocal imaging, animals were deeply anesthetized using isoflurane, followed by intracardial perfusion of phosphate buffered saline (PBS) and 4% PFA in PBS (pH 7.35). Brains were harvested immediately after perfusion. The tissues were then fixed for 8 h in 4% PFA in PBS and cryopreserved by overnight incubations in 20% sucrose. Tissues were embedded in OCT compound (Fisher HealthCare) and sectioned. For immunohistochemistry analysis of different brain regions, 30 μm sections were collected with a cryostat and stained as floating sections. For antigen retrieval, sections were incubated in Sodium Citrate Buffer (10 mM, 0.05% Tween 20, pH 6.0) at 75 °C for 15 min. Sections were then blocked for 20 min in 10% goat serum or donkey serum in PBS with 0.3% Triton X-100, followed by incubation with primary antibody dilutions in blocking buffer at 4 °C overnight. Primary antibodies were used at the following concentrations: anti-GFP (ab13970, abcam, 1:1000), α-Sox9 (Ab5535, Millipore, 1:1000), and α-OMP (019-22291, Wako, 1:8000). Secondary antibody incubation (647 α-goat, #A21477, Lot 2045332, Life Technologies, 1:500;647 α-chicken, #A-21449, ThermoFisher, 1:500; 647 α-rabbit, #A21244, Lot 1990307, LifeTechnologies, 1:500) was performed in the blocking solution with 0.1% Triton X-100 at room temperature for 1 hr. Sections were washed between incubations with PBS containing 0.1% Triton X-100. DAPI was included in the penultimate wash. Imaging for morphological analysis was performed using Zeiss Imager M2 and Z1 fluorescence microscope equipped with a Zeiss AxioCam camera. Analysis was performed using ZEN 3.1 and ImageJ software.

To measure astrocyte morphology, fluorescent images were acquired using a Zeiss LSM 880 laser scanning confocal microscope with 63× oil immersion objective with frame size at 1024 × 1024 and bit depth at 12. Serial images in the z-axis were taken at an optical step of 1 mm, with overall z-axis range encompassing the whole section. Images were imported to Imaris Bitplane software, and only astrocytes with their soma between the z-axis range were chosen for further analysis. We performed 3D surface rendering (Figs. 1e, h, 6a, and S3a) using the Imaris Surface module. Morphological analysis was performed using the Imaris Filament module. Astrocyte branches and processes were outlined by Autopath with starting point set at 8 mm and seed point set at 0.5 mm. Data were generated from 3 brain sections per region per mouse with 3 mice per genotype. For viral-mediated KO or KD animals, only viral infected cells were selected for the morphology analysis. To analyze number of astrocytes, fluorescent images were acquired using a Zeiss LSM 880 laser scanning confocal microscope with 20× objective. Cell numbers were quantified by the QuPath software Cell Detection function^75^.

Olfactory marker protein (OMP) was quantified using Imaris 9.7 from 40 μm z-stacks. Briefly, a mask was drawn to include only the glomerular layer of the olfactory bulb within each section. OMP was detected by thresholding fluorescence intensity values within this mask. A 3D surface object encompassing the thresholded OMP was created. This surface was then normalized using either total number of distinct glomeruli (counted by the researcher) or by DAPI+ cells quantified by Imaris within the glomerular layer mask. Normalized values were then graphed using GraphPad Prism version 9.1

#### Calcium imaging

In vivo wide-field calcium imaging was performed in anesthetized (urethane, 1.2 µm/kg) head-fixed animals (8–14 weeks old) through thinned skulls 2–3 weeks after viral injection. Animals were kept warm with a portable heating pad and a local anesthetic (bupivicane, 0.5%) was applied to the incision areas. Sensory evoked responses were acquired with a Leica M205FA microscope at 6x magnification, a GFP filter set and a Leica FL6000 fluorescence light source. Optical signals were recorded for 10 s per trial at 464 × 346 pixel resolution. A CCD camera (DFC360 FX, Leica) captured images at a frame rate of 10 Hz and videos were digitized at 12 bits using Leica Application Suite software. Odorants were presented using a custom-made olfactometer (stimulus duration, 3 s) with separate lines and were presented as ~1% saturated vapor, adjusted for vapor pressure. The odors were presented in a random order with at least three trials per odorant at interstimulus intervals ≥60 s.

For two-photon calcium imaging, mice were deeply anesthetized with isoflurane and then perfused with cold artificial cerebrospinal fluid (ACSF, in mM:125 NaCl, 25 glucose, 25 NaHCO_3_, 2.5 KCl, 2 CaCl_2_, 1.25 NaH_2_PO_4_, and 1 MgCl_2_, pH 7.3, 310–320 mOsm). The brain was dissected, embedded in low melting point agarose, and placed in an ice-cold sucrose-based cutting solution (in mM: 87 NaCl, 2.5 KCl, 1.6 NaH_2_PO_4_, 25 NaHCO_3_, 75 sucrose, 10 glucose, 1.3 ascorbic acid, 0.5 CaCl_2_, 7 MgCl_2_). 300 µm thick coronal olfactory bulb slices were sectioned on a vibratome (VT1200, Leica). Slices were then recovered in oxygenated ACSF (37 °C) for 15 min and allowed to acclimate to room temperature for at least 15 min before imaging.

We recorded calcium traces using a two-photon resonant microscope equipped with a Chameleon Ultra (II) Ti-sapphire laser (Coherent) tuned to 900 nm through a 20×, 1.0 NA Zeiss objective. Calcium activity was typically sampled at ~1 Hz. Optical signals were recorded for ~5 min per trial at 1024 × 1024 pixel resolution. We recorded data from astrocytes at depths of ~30 µm below the surface. All multiphoton imaging experiments were performed within 2–4 h of slicing.

For ex vivo wide-field imaging, slices were prepared as described above for two-photon imaging. Optical signals were recorded for 10 s per trial at 1392 × 1040 resolution with 4 × 4 binning. A CCD camera (QIcam Fast 1934, QImaging) capture images at a frame rate of ~35 Hz and videos were digitized at 8 bit using Q Capture Pro 7. Glutamate (500 mM in ACSF) was presented through puff application using a FemtoJet 4× with Pi = 1.5 psi and *P*_c_ = 0.07 psi for 500 ms. Data were analyzed in ImageJ by measuring the intensity of a region of interest (ROI) exhibiting a response from glutamate and in MATLAB with the Curve Fitting Toolbox.

#### Whole-cell patch clamp electrophysiology

Animals were deeply anesthetized with halothane. After decapitation, the brain was quickly excised from the skull and submerged in an ice‐cold cutting solution (in mM: 130 NaCl, 24 NaHCO_3_, 1.25 NaH_2_PO_4_, 3.5 KCl, 1.5 CaCl_2_, 1.5 MgCl_2_, and 10 D(+)‐glucose, pH 7.4). 300 μm sagittal slices were cut using a vibratome (DSK Linear Slicer, Kyoto, Japan) with a blade (DORCO, Seoul, Korea) and transferred to oxygenated ACSF solution (in mM: 130 NaCl, 24 NaHCO_3_, 1.25 NaH_2_PO_4_, 3.5 KCl, 1.5 CaCl_2_, 1.5 MgCl_2_, and 10 D(+)‐glucose, pH 7.4).

Whole‐cell recordings were made from mitral, granule cells, and astrocytes somata located in the olfactory bulb. For action potential measurement, mitral and granule cells were patched with an internal solution (in mM): 140 K-gluconate, 10 HEPES, 7 NaCl, and 2 MgATP adjusted to pH 7.4 with CsOH and their membrane potential were set at −60 mV. The current was injected from 0 to 80 pA for 50 and 500 ms. For passive conductance measurement, astrocytes were patched with an internal solution (in mM): 140 K-gluconate, 10 HEPES, 7 NaCl, and 2 MgATP adjusted to pH 7.4 with CsOH and their membrane potential were set at −60 mV. The voltage was injected from −120 to 120 mV for 1 s. For miniature excitatory postsynaptic currents (mEPSCs), M/T cells in oxygenated ACSF containing 0.5 µM TTX and 20 µM Bicuculline were patched with an internal solution (in mM): 135 CeMeSO_4_, 8 NaCl, 10 HEPES, 0.25 EGTA, 1 Mg-ATP, 0.25 Na_2_-GTP, and 30 QX-314 adjusted to pH 7.2 with CsOH. For miniature inhibitory postsynaptic currents (mIPSCs), M/T cells in oxygenated ACSF containing 0.5 µM TTX, 50 µM AVP, and 20 µM CNQX were patched with an internal solution (in mM): 135 CsCl, 4 NaCl, 0.5 CaCl_2_, 10 HEPES, 5 EGTA, 2 Mg-ATP, 0.5 Na_2_-GTP, and 30 QX-314 adjusted to pH 7.2 with CsOH. mEPSCs and mIPSCs were recorded in the presence of TTX. Pipette resistance was typically 4–6 MΩ. Electrical signals were digitized and sampled at 50 μs intervals with Digidata 1550B and Multiclamp 700B amplifier (Molecular Devices) using pCLAMP 10.4 software. Data were filtered at 2 kHz.

For astrocyte glutamate transporter current recording, *Sox9*^*fl/fl*^ and *Sox9*^*+/+*^ mice with AAV-GFAP-iCre-P2a-TurboRFP injection in olfactory bulbs were used. Slices were prepared as described above for two-photon. RFP + astrocytes were patched in whole-cell voltage clamp configuration with an internal solution (in mM): 120 K-Gluconate, 20 HEPES, 10 EGTA, 2 MgATP, and 0.2 NaGTP adjusted to pH 7.2 and 295 mOsm. Pipette resistance was 6–8 MΩ. ACSF included CNQX (20 µM), APV (50 µM), BIC (10 µM), and TTX (1 µM). Astrocytes (~50–75 µm deep from surface) were held at −80 mV and continually monitored for changes in access resistance. Recordings with a change in access resistance of more than 25% were excluded. To evoke transporter currents, glutamate (500 mM in ACSF) was presented through puff application perpendicular to flow of ACSF using a FemtoJet 4x with _Pi_ = 0.2–0.8 psi and P_c_ = 0–0.07 psi for 500 ms at 50–100 μm from the patched astrocyte soma in the same plane. Glass pipettes for delivering the puff solution had resistances of 1–2 MΩ. In total 10–20 trials were recorded at baseline then TBOA (100 µM) was applied to block glutamate transporter currents. Transporter currents were quantified as the average of the trials after complete wash on of TBOA subtracted from the average of the trials before TBOA application. Kinetics of the puff-evoked transporter currents were quantified as the fast time constant of a biexponential fit of the subtracted current.

#### Single cell RNA-seq analysis

For single cell sequencing analysis, we have used our previously published dataset with the accession number GEO: GSE121891^35^. Matrices from each experiment were imported into Seurat (version 2.2.1)^76^. The minimum gene per cell threshold was set to 200 and maximum gene per cell threshold was set to 2500 for inclusion into the final digital expression matrix. Immediate early genes were removed from differentially expressed genes^77^ before canonical correlation analysis (CCA) which was used to scale and merge datasets from each experimental batch to account for batch effects^76^. Two-dimensional visualization of the multi-dimensional data set was done with t-SNE. Differential expression of the individual clusters was performed using the likelihood-ratio test for single cell gene expression (Seurat FindMarkers function, default parameters).

#### Cell dissociation and FAC-sorting of astrocytes

*Sox9*^*fl/fl*^ and *Sox9*^*+/+*^ mice with AAV-GFAP-iCre-P2a-TurboRFP injection in olfactory bulbs were used to isolate astrocytes by fluorescence-activated cell sorting (FACS). Four weeks after the injection, olfactory bulbs from the mice were dissociated following published protocols^51^ with slight modifications. Briefly, the olfactory bulbs from 6 mice (3 control and 3 KO males) were dissected and digested for 15 min at 37 °C with 0.5 mL papain solution (1x EBSS (Worthington), 230 U/ml DNase (Worthington), 25 U/ml papain (Worthington)) in 1.5 mL microcentrifuge tubes on the thermomixer with 1400 rpm shaking for agitation. After digestion, the tissue was neutralized with DMEM/F12 + 10% FBS solution (ThermoFisher) and centrifuged at RT at 4200 × *g* for 5 min. The resultant pellet was washed by PBS and re-suspended in FACS buffer (Leibovitz’s L-15 Medium with 0.01 M HEPES, 0.0025% DNaseI), and filtered with tubes with cell-strainer caps (FALCON). FACS was performed in a FACSAria I (BD Bioscience, 100-μm nozzle and 20-p.s.i. setting), and astrocytes were separated by TurboRFP. FAC-sorted cells were collected in RLT lysis buffer (QIAGEN) with 1% beta-mercaptoethanol.

#### Bulk RNA extraction, library preparation, and sequencing

RNA was extracted from FACS pelleted cells using RNeasy Micro Kit (Cat. No. 74004, QIAGEN). RNA integrity (RIN ≥ 8.0) was confirmed using the High Sensitivity RNA Analysis Kit (DNF-472-0500, Agilent formerly AATI) on a 12-Capillary Fragment Analyzer. Illumina sequencing libraries with 8-bp single indices were constructed from 10 ng total RNA using the Trio RNA-Seq System (0507-96, NuGEN). The resulting libraries were validated using the Standard Sensitivity NGS Fragment Analysis Kit (DNF-473-0500, Agilent formerly AATI) on a 12-Capillary Fragment Analyzer. Equal concentrations (2 nM) of libraries were pooled and subjected to sequencing of approximately 40 million reads per sample using the High Output v2 kit (FC-404-2002, Illumina) on a NextSeq550 following the manufacturer’s instructions.

#### qRT-PCR

Real time quantitative PCR was carried out using Powerup SYBR green master mix (A25742, ThermoFisher) and a Quant Studio 3 real-time thermo cycler (Applied Biosystems). Primer sets listed in Supplementary Table 1 were used to specifically amplify their respective targets. Each sample was run in triplicate and with negative control (without cDNA). Sample amplification curves were filtered based on internal quality control parameters for specificity and passive reference ROX dye was used normalize for optical path differences. Data analysis was done using the ∆∆C_T_ method^78^ with all data normalized to ActinB or GAPDH expression.

#### Three compartment place preference assay

Mice received viral injections at least 4 weeks prior to behavioral experiments. Mice were acclimated and familiarized to the testing chamber for 5 min each day for three days prior to testing. For odor detection threshold, odor concentrations were tested for 2 min each in ascending order of concentration with 1 min between each. For odor discrimination, a habituation-dishabituation assay was used in which mice were habituated to an odor for 3 trials before being exposed to a novel odor. Each trial lasted 2 min with 1 min between each trial. Video was captured with a Doric GigE Behavior Tracking Camera (BTC_GigE_CO) and position tracking was performed with semiautomated analysis with OptiMouse through MATLAB. Analysis of these behavior data were performed blind and objectively through OptiMouse software in MATLAB.

### Quantification and statistical analysis

For wide-field calcium imaging, all data analysis was performed with custom MATLAB scripts and processed in ImageJ. Initial data processing included averaging trials and visually aligning the region of interest (dorsal olfactory bulb). Δ*F* maps were created by temporally averaging the entire 3 s odor stimulation period and subtracting the temporal average of the 2 s immediately preceding odor onset. Δ*F/F* maps were additionally normalized by the first five frames of the averaged trial. There was no appreciable photobleaching of the GCaMP3 signals during the experiments. For quantification, ROIs approximately the size of a glomerulus were set over activated regions with at least 3 ROIs per odorant. For two-photon data processing, all analysis was performed in custom MATLAB scripts.

For two-photon imaging of astrocyte microdomains, ROIs were generated posthoc with GECIquant plugin in FIJI and time analysis was performed with custom MATLAB scripts.

Sequencing files were de-multiplexed and the resulting fastq files were merged. Quality control was performed using fastQC (v0.10.1) and MultiQC (v0.9). Reads were mapped to the mouse genome mm10 assembly using STAR (v2.5.0a). BAM files were normalized for sequencing depth using samtools (v.1.3). Bioconductor packages GenomicAlignments (v1.16.0) and GenomicFeatures (v1.32.2) were used to build count matrices. Differential expression and normalized count values were determined using DESeq2 (v1.20.0) in R (v 3.5.2). GO analyses were performed by Enrichr website.

### Reporting summary

Further information on research design is available in the Nature Research Reporting Summary linked to this article.

## Supplementary information

Supplementary Information

Reporting Summary

## Data Availability

The datasets generated during and/or analyzed during the current study that are necessary to interpret, verify, and extend the research are provided in Figs. 1–6, Supplementary Figs. 1–6, Supplementary Table 1, and the Source Data file. The single-cell RNA sequencing data used in this study are available in the GEO database under accession code GSE121891. The FACS-sorted RNA sequencing data generated in this study have been deposited in the GEO database under accession code GSE180841. All other additional information and reagents will be available from the corresponding author upon reasonable request. Source data are provided with this paper.

## Source data

Source Data

## Code availability

The code used to analyze data are freely available on Github (github.com/arenkiellab).
