# Supplementary material for: Olfactory bulb astrocytes mediate sensory circuit processing through Sox9 in the mouse brain
Source: Nat Commun. 2021 Sep 1;12:5230. doi: 10.1038/s41467-021-25444-3 (PMC8410770; doi:10.1038/s41467-021-25444-3)
Supplement: Supplementary file 1 — Supplementary Information [file 41467_2021_25444_MOESM1_ESM.pdf]

# Ung et. al.Supplementary Figure 1

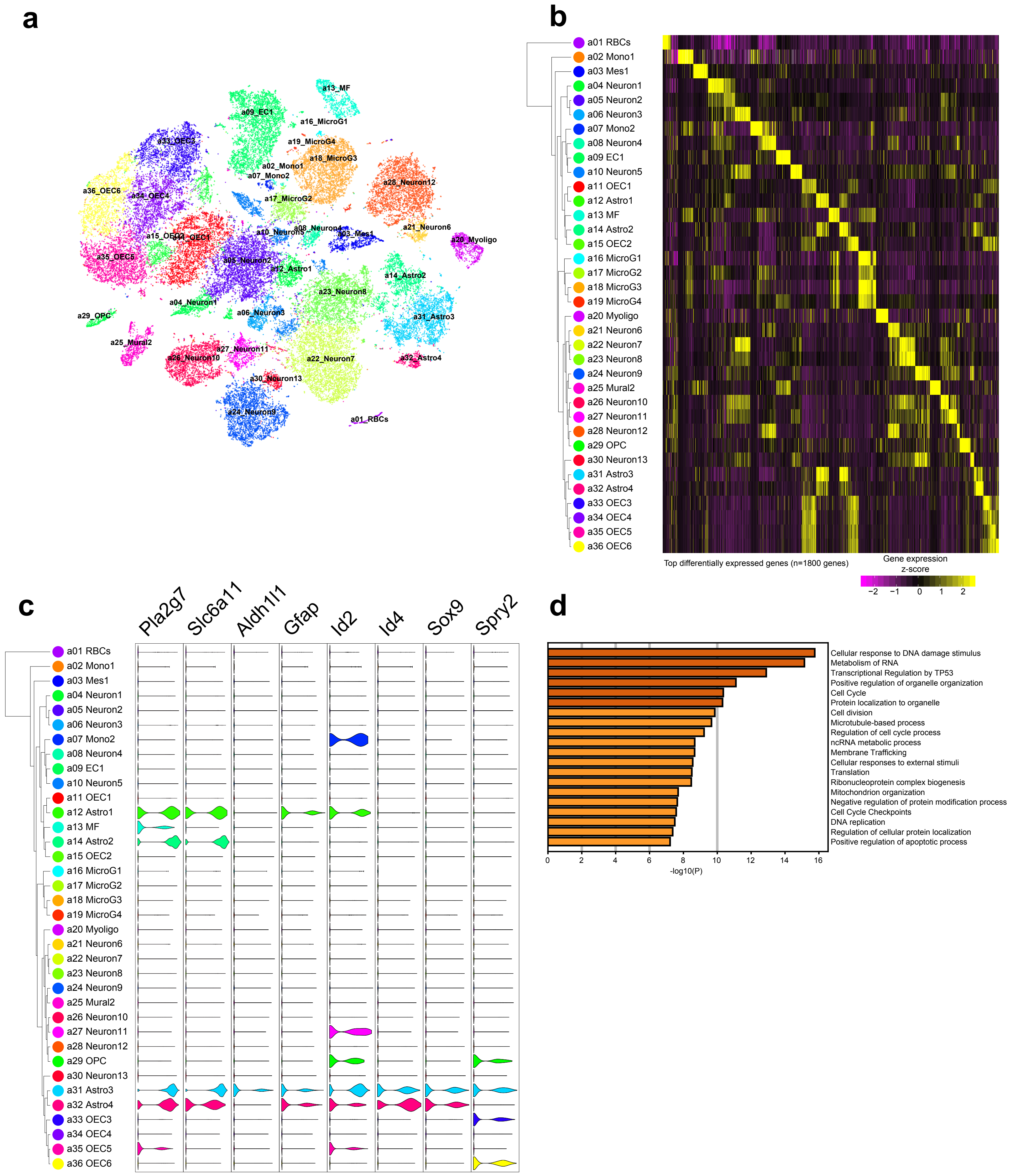

**Supplemental Figure S1. Analysis of RNA-seq reveals gene expression patterns based on cluster identity.**

(a) Cellular composition of the OB was visualized using t-distributed stochastic neighbor embedding (t-SNE). Single cell sequencing allowed unbiased clustering of cell populations in the OB, revealing 36 distinct populations.

(b) Heat map of top 50 enriched genes in each cluster from single-cell sequencing

(c) Graph showing all detected clusters with violin plots for expression of selected marker genes.

(d) GO analysis from top genes enriched in astrocyte cluster 3 expressing Aldh1L1.

# Ung et. al.Supplementary Figure 2

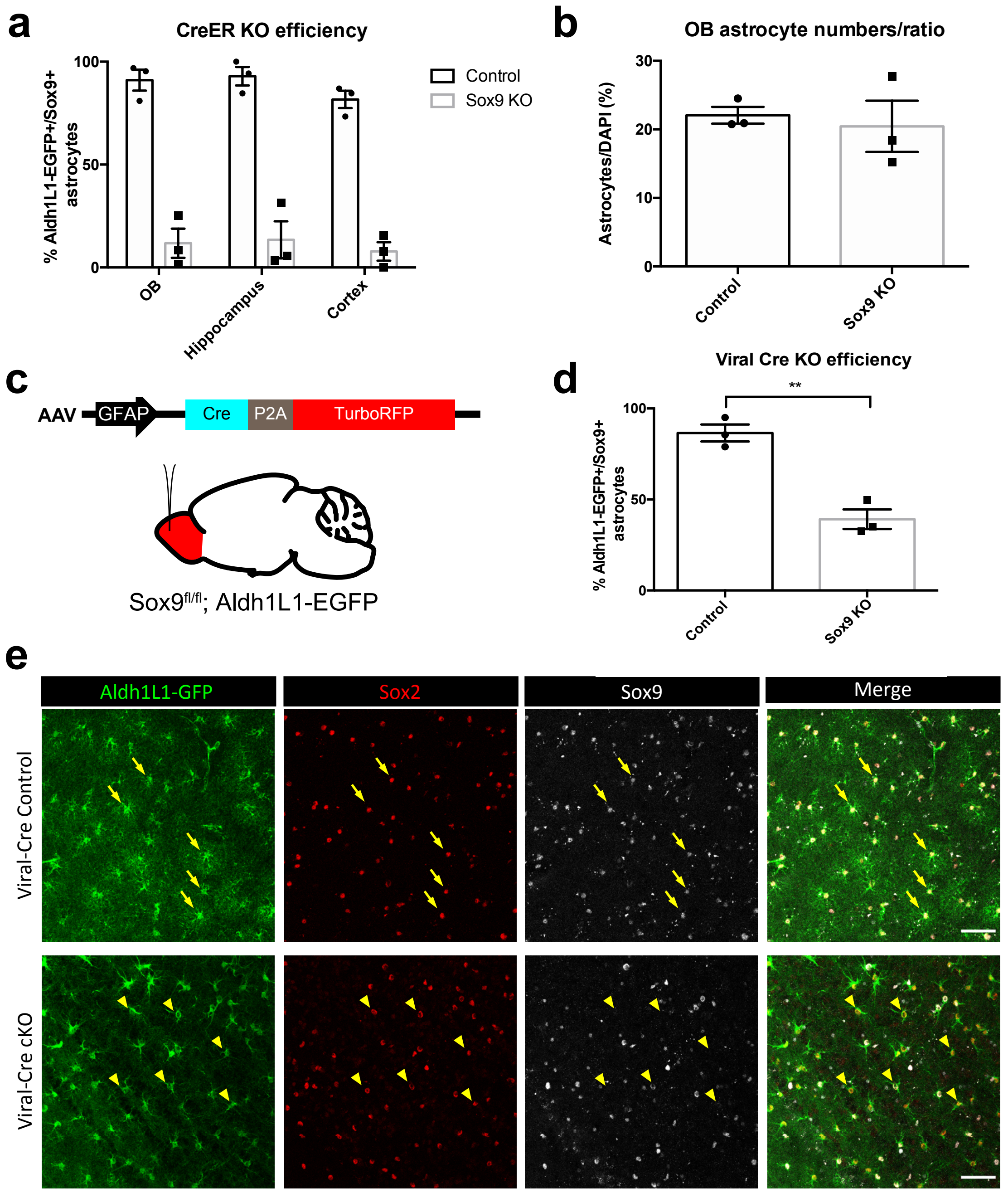


**Supplemental Figure S2. Virally introduced Cre allows for local, efficient knockout of Sox9**
(a) Quantification of KO efficiency in CAG-CreER; Sox9fl/fl from N = 3 mice per group; Aldh1L1-EGFP mice revealed that tamoxifen treatment induced high efficiency knockout of Sox9 in EGFP+ astrocytes. Data are presented as mean values ± SEM.
(b) Quantification of EGFP+ cells relative to DAPI revealed no changes in astrocyte numbers after Sox9 knock out from N = 3 mice per group. Data are presented as mean values ± SEM.
(c) Schematic of virally-induced Sox9 KO.
(d-e) Quantification of KO efficiency in Sox9fl/fl from N = 3 mice per group; Aldh1L1-EGFP mice injected with AAV-GFAP-iCre-P2a-TurboRFP revealed that viral delivery of Cre generates efficient knockout of Sox9 in EGFP+ astrocytes. **p =0.0027, Unpaired two-sided Student's t-test. Data are presented as mean values ± SEM. Scale bar, 50 μm.

Ung et. al.Supplementary Figure 3

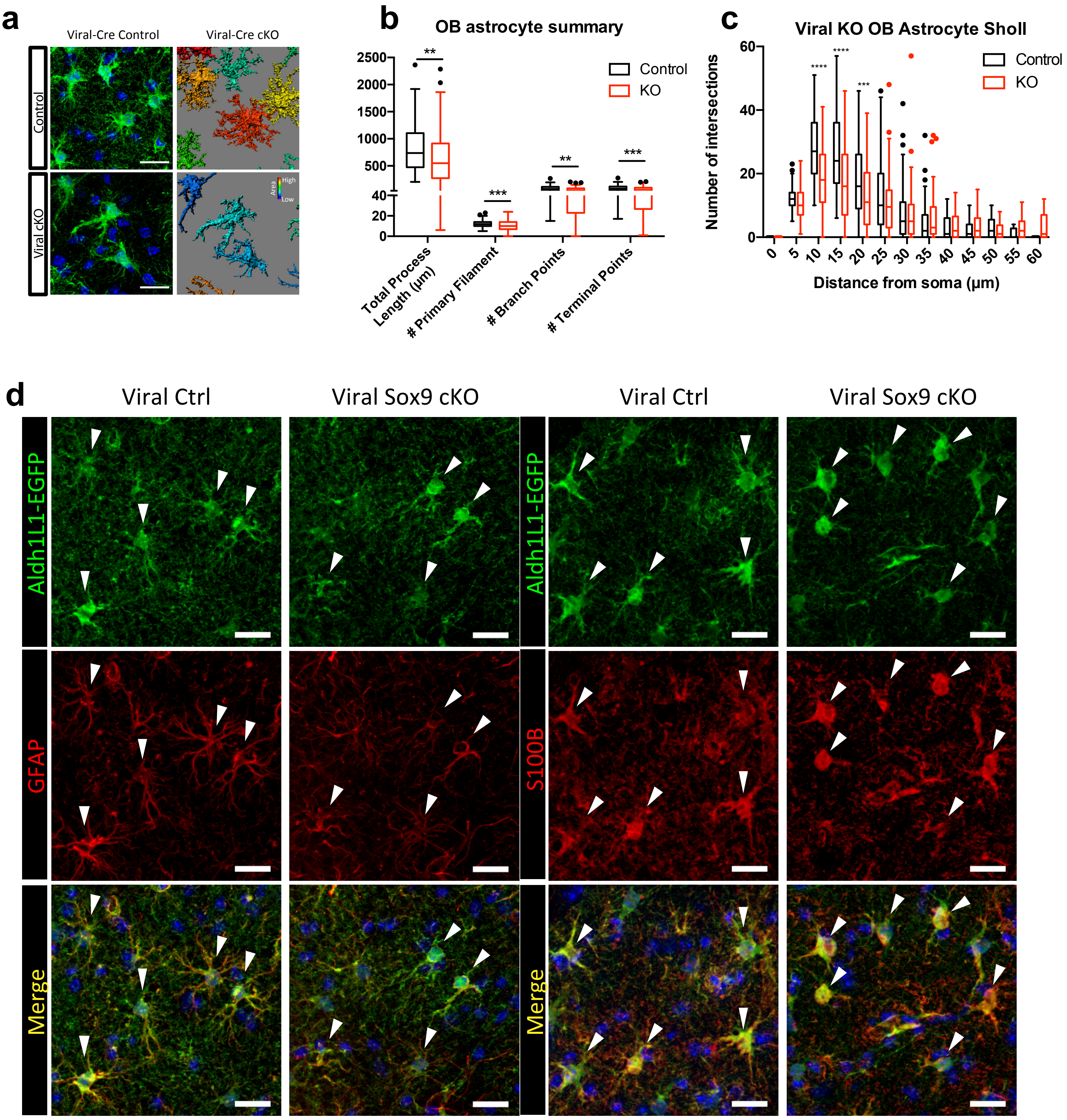

**Supplemental Figure S3. Viral KO of Sox9 in OB astrocytes results in loss of morphological complexity**

(a) High-resolution imaging and surface rendering of astrocytes in OB shows a decrease in morphological branching in Sox9 KO vs controls.

(b-c) Quantification of (a). n = 87 cells (control) and n = 94 cells (Sox9 KO) from N = 4 mice, respectively. $p = 0.0021$, 0.0004, 0.0010, and 0.0008 for total process length, # primary filaments, # branch points, and # terminals points, respectively; ***$p = 0.0004$, ****$p < 0.0001$ for Sholl analysis. 2-way ANOVA with Sidak's multiple comparison correction. Data are presented as box plots displaying interquartile range and median with Tukey whiskers. Scale bar, 20 µm.

(d) Staining with GFAP and S100B confirms loss of morphological complexity with Sox9 KO in astrocytes. N = 3 mice. Scale bar, 20 µm.

Ung et. al.Supplementary Figure 4

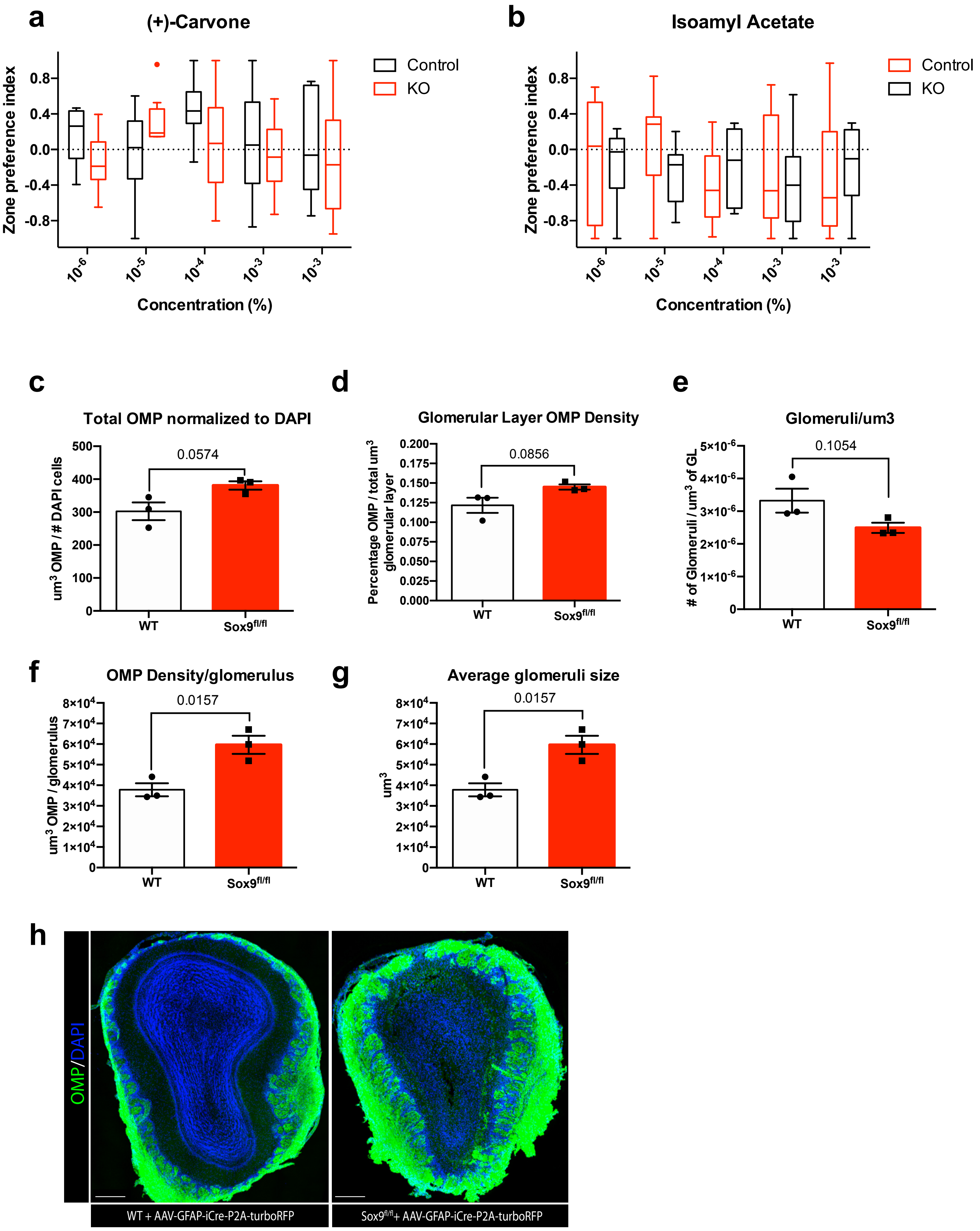

**Supplemental Figure S4. Odor preference is not indicative of sensitivity changes.**
(a) Mice with Sox9 knocked out of OB astrocytes may be more sensitive to (+)-Carvone than control mice. Data are presented as box plots displaying interquartile range and median with Tukey whiskers. Two-way RM ANOVA with Bonferroni's multiple comparison's test (main effect of group, $F(1,16) = 0.8905$, $p = 0.3594$; group X concentration interaction $F(4,64) = 2.190$, $p = 0.0800$). Mice spent more time in the chamber investigating the odor upon detection of (+)-Carvone.
(b) Mice with Sox9 knocked out of OB astrocytes showed no significant change in odor sensitivity to isoamyl acetate, a non-preferred odor, compared to control mice. Data are presented as box plots displaying interquartile range and median with Tukey whiskers. Two-way RM ANOVA with Bonferroni's multiple comparison's test (main effect of group, $F(1, 16) = 0.04623$, $p = 0.8325$; group X concentration interaction $F(4,64) = 1.270$, $p = 0.2911$).
(c-g) Quantification of olfactory marker protein (OMP) in the glomerular layer of OBs with Sox9 KO of astrocytes or in control OBs from N = 3 mice per group. Two-sided Student's t-test. Data are presented as mean values ± SEM.
(h) Representative image of OMP staining in control OB (left) vs OB with Sox9 KO of astrocytes (right). Scale bars, 300 μm (left) and 200 μm (right).

# Ung et. al.Supplementary Figure 5

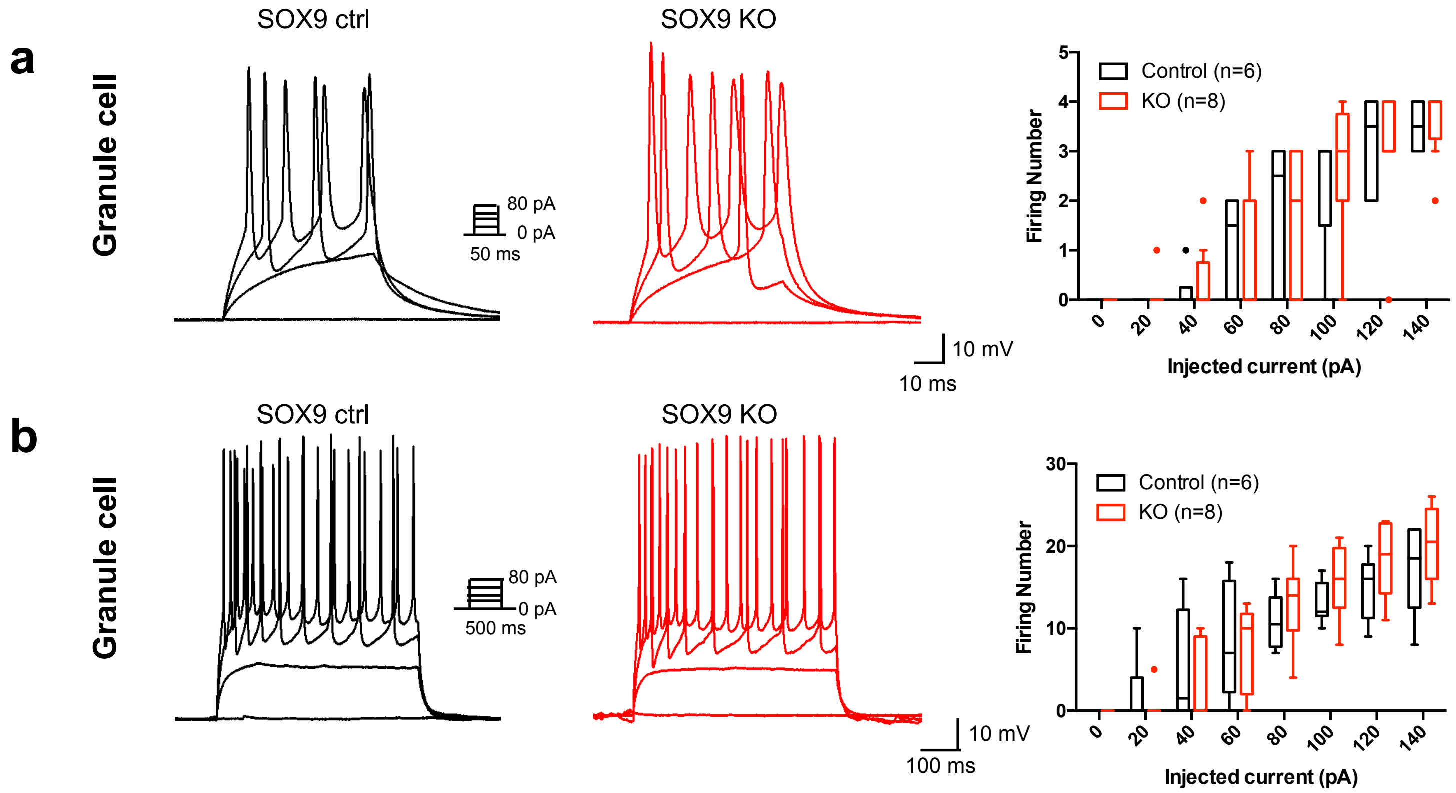


**Supplemental Figure S5. Electrophysiological recordings showed no changes to intrinsic properties granule cells.**
(a-b) Whole-cell patch clamp electrophysiology of granule cells revealed no changes in firing rate from stepped current injections (top, 50ms; bottom, 500ms) with Sox9 KO in astrocytes vs control astrocytes. 2-way ANOVA with Sidak's multiple comparison correction. Data are presented as box plots displaying interquartile range and median with Tukey whiskers.

# Ung et. al.Supplementary Figure 6

**a**

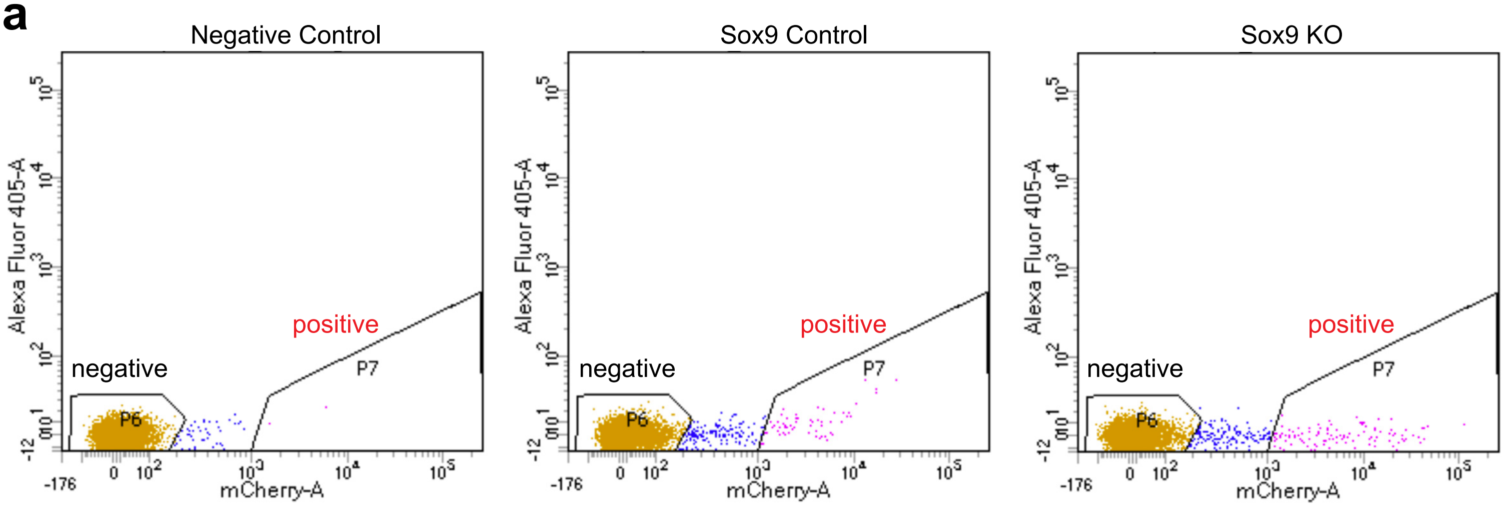


**b**

| Population | % Total | SEM |
|---|---|---|
| control-neg | 62.00 | 0.12 |
| control-pos | 0.90 | 0.32 |
| KO-neg | 58.10 | 0.20 |
| KO-pos | 1.27 | 0.13 |

**c**

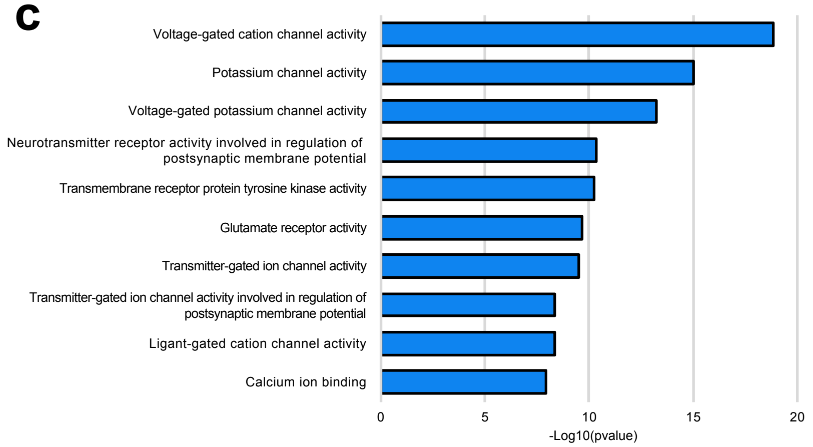


**d**

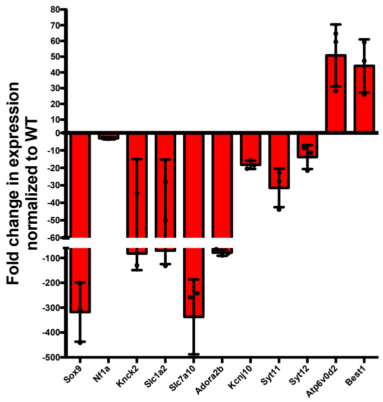


**e**

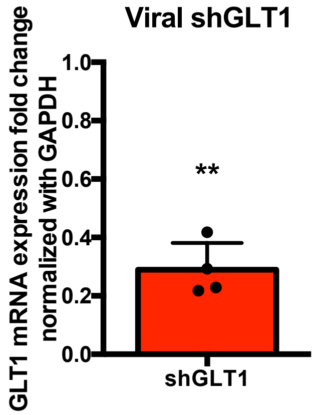


**Supplemental Figure S6. GO analysis of Sox9 results in upregulation and downregulation of genes associated with many cellular functions.**

(a) FACS plots show negative and positive populations from negative control olfactory bulb (left), Sox9 control olfactory bulb (middle), and Sox9 cKO olfactory bulb (right).

(b) Table shows percentage of negative and positive populations in total cells.

(c) Downregulated genes from Sox9 deletion revealed GO terms associated with a range of cellular functions.

(d) qRT-PCR of select genes from RNAseq verifying changes in expression levels of mRNA transcripts. Data are presented as mean values ± SEM.

(e) GLT1 mRNA normalized to GAPDH was significantly reduced in shGLT1 injected tissue compared to shScramble. **$p < 0.01$. Two-sided Student's t-test. Data are presented as mean values ± SEM.

Ung et. al.Supplementary Table 1

| Gene | Forward primer | Reverse primer |
|---|---|---|
| Slc6a1 | GAAAGCTGTCTGATTCTGAGGTG | AGCAAACGATGATGGAGTCCC |
| Kcnk2 | CCGAGGCTCTCATTCTCCTCA | AGGACGACCACCAGGAAAATC |
| Sypl2 | CATGCGGAGTCCCGGATTC | CAGAGTTCTGGAAAGGTTACACC |
| Adora2b | AGCTAGAGACGCAAGACGC | GTGGGGGTCTGTAATGCACT |
| Adora1 | TGTGCCCGGAAATGTACTGG | TCTGTGGCCCAATGTTGATAAG |
| Slc7a10 | GGGTTTGGCCCTCTTCGTC | GACATAGGCGTAGTCCCCAC |
| Kcnj10 | GTCGGTCGCTAAGGTCTATTACA | GGCCGTCTTTCGTGAGGAC |
| Kcnn3 | TGTTGCACTCTTCTCCCACG | GGTCATTGAGATTTAGCTGGCT |
| Slc1a2 | GCACGAGAGCTATGGTGTATTAC | GTTTGGGATTACCTGGGTGGA |
| Atp6v0d2 | CAGAGCTGTACTTCAATGTGGAC | AGGTCTCACACTGCACTAGGT |
| P2ry14 | AGCAGATCATTCCCGTGTTGT | AGCCACCACTATGTTCTTGAGA |
| Adora3 | AAGGTGAAATCAGGTGTTGAGC | AGGCAATAATGTTGCACGAGT |
| Best1 | ACACAACACATTCTGGGTGC | CGCAAAGTACACACCTCATTCA |
| Syt11 | GAGATCACAAATATACGCCCCAG | GCAGCACGTCCACACAAAG |
| Syt12 | GGAGGTGGGTGTCTATGCTG | CTGGTCATTCCAAGGAGGGAC |
| Nfia | GCCCGGCAGTTATGTATTCTC | GGTTGAACCATGTGTAGGCGA |
| ActinB | TAACAGTCCGCCTAGAAGCA | GCAAGCAGGAGTACGATGAG |

**Supplementary Table 1. qRT-PCR primers used for verification of RNA-seq data**
