# Supplementary material for: Olfactory bulb astrocytes mediate sensory circuit processing through Sox9 in the mouse brain
Source: Nat Commun. 2021 Sep 1;12:5230. doi: 10.1038/s41467-021-25444-3 (PMC8410770; doi:10.1038/s41467-021-25444-3)
Supplement: Supplementary file 2 — Reporting Summary [file 41467_2021_25444_MOESM2_ESM.pdf]

nature research

Corresponding author(s): Benjamin R. Arenkiel

Last updated by author(s): Aug 6, 2021

Reporting Summary

Nature Research wishes to improve the reproducibility of the work that we publish. This form provides structure for consistency and transparency in reporting. For further information on Nature Research policies, see our Editorial Policies and the Editorial Policy Checklist.

Statistics

For all statistical analyses, confirm that the following items are present in the figure legend, table legend, main text, or Methods section.

n/a	Confirmed	
☐	☒	The exact sample size (*n*) for each experimental group/condition, given as a discrete number and unit of measurement
☐	☒	A statement on whether measurements were taken from distinct samples or whether the same sample was measured repeatedly
☐	☒	The statistical test(s) used AND whether they are one- or two-sided<br>*Only common tests should be described solely by name; describe more complex techniques in the Methods section.*
☒	☐	A description of all covariates tested
☐	☒	A description of any assumptions or corrections, such as tests of normality and adjustment for multiple comparisons
☐	☒	A full description of the statistical parameters including central tendency (e.g. means) or other basic estimates (e.g. regression coefficient) AND variation (e.g. standard deviation) or associated estimates of uncertainty (e.g. confidence intervals)
☐	☒	For null hypothesis testing, the test statistic (e.g. *F*, *t*, *r*) with confidence intervals, effect sizes, degrees of freedom and *P* value noted<br>*Give P values as exact values whenever suitable.*
☒	☐	For Bayesian analysis, information on the choice of priors and Markov chain Monte Carlo settings
☒	☐	For hierarchical and complex designs, identification of the appropriate level for tests and full reporting of outcomes
☒	☐	Estimates of effect sizes (e.g. Cohen's *d*, Pearson's *r*), indicating how they were calculated

Our web collection on statistics for biologists contains articles on many of the points above.

Software and code

Policy information about availability of computer code

Data collection	Quant Studio 3 qPCR Data Analysis Software, pCLAMP, SnapGene, ZEN3.1, FIJI, QuPath, Leica Application Suite, Q Capture Pro 7, RStudio 1.4, fastQC (v0.10.1), MultiQC (v0.9), STAR (v2.5.0a), samtools (v1.32.2), Genomic Alignments (v1.16.0), GenomicFeatures (v1.32.2), DESeq2 (v1.20.0)
Data analysis	Statistical analyses were performed in GraphPad Prism 9.1. Automated ROI detection for calcium imaging was performed with GECIquant in FIJI 2.1.0/1.53c and analysis was performed using custom MATLAB (R2018b) script. Automated detection for mouse behavior was performed with OptiMouse in MATLAB. Single cell RNAseq analysis was performed in Seurat (version 2.2.1). Two-dimensional visualization of the multi-dimensional data set was done with t-SNE.

For manuscripts utilizing custom algorithms or software that are central to the research but not yet described in published literature, software must be made available to editors and reviewers. We strongly encourage code deposition in a community repository (e.g. GitHub). See the Nature Research guidelines for submitting code & software for further information.

Data

Policy information about availability of data

All manuscripts must include a data availability statement. This statement should provide the following information, where applicable:

- Accession codes, unique identifiers, or web links for publicly available datasets
- A list of figures that have associated raw data
- A description of any restrictions on data availability

The datasets generated during and/or analysed during the current study that are necessary to interpret, verify, and extend the research are provided in Figs. 1-6, Supplmentary Figs 1-6, Supplementary Table 1, and the Source Data file. The single-cell RNA sequencing data used in this study are available in the GEO database under accession code GSE121891 [https://www.ncbi.nlm.nih.gov/geo/query/acc.cgi?acc=GSE121891]. The FACS-sorted RNA sequencing data generated in this

study have been deposited in the GEO database under accession code GSE180840 [https://www.ncbi.nlm.nih.gov/geo/query/acc.cgi?acc=GSE180841].

Field-specific reporting

Please select the one below that is the best fit for your research. If you are not sure, read the appropriate sections before making your selection.

☒ Life sciences ☐ Behavioural & social sciences ☐ Ecological, evolutionary & environmental sciences

For a reference copy of the document with all sections, see nature.com/documents/nr-reporting-summary-flat.pdf

Life sciences study design

All studies must disclose on these points even when the disclosure is negative.

Sample size	No estimates of statistical power were performed before experiments; animal numbers were minimized to conform to ethical guidelines while accurately measuring neuronal (electrophysiology) or behavioral responses and our sample sizes are similar to those reported in previous publications (Quast et al., 2015, Breton-Provencher et. al., 2009)
Data exclusions	For electrophysiological recordings, recordings with changes in access resistance of more than 25% were excluded. This exclusion criteria was pre-established because large changes in access resistance affects the quality of voltage clamp recordings.
Replication	Olfactory behavior tasks were performed on multiple sets of odorant stimuli on different days such that behavior can be generalized. Experiments were performed once per odor for a set of 3 different odors with all replication attempts successful.
Randomization	Littermate animals were randomly selected into the control or experimental groups.
Blinding	Analysis was performed blind to manipulation, including in data which automated analyses were performed.

Reporting for specific materials, systems and methods

We require information from authors about some types of materials, experimental systems and methods used in many studies. Here, indicate whether each material, system or method listed is relevant to your study. If you are not sure if a list item applies to your research, read the appropriate section before selecting a response.

Materials & experimental systems

n/a	Involved in the study	
☐	☒	Antibodies
☒	☐	Eukaryotic cell lines
☒	☐	Palaeontology and archaeology
☐	☒	Animals and other organisms
☒	☐	Human research participants
☒	☐	Clinical data
☒	☐	Dual use research of concern

Methods

n/a	Involved in the study	
☒	☐	ChIP-seq
☐	☒	Flow cytometry
☒	☐	MRI-based neuroimaging

Antibodies

Antibodies used	Primary: ck-anti-GFP (ab13970, abcam), rb-anti-Sox9 (Ab5535, Millipore), anti-OMP (019-22291, WAKO)<br>Secondary: 647 anti-goat (#A21447, Lot 2045332, Life Technologies), 647 anti-chicken (#A-21449, ThermoFisher), 647 anti-rabbit (#A21244, Lot 1990307, LifeTechnologies)
Validation	anti-GFP references: https://www.abcam.com/gfp-antibody-ab13970.html<br><br>anti-Sox9 references: https://www.emdmillipore.com/US/en/product/Anti-Sox9-Antibody,MM_NF-AB5535#anchor_REF<br><br>anti-OMP references: https://labchem-wako.fujifilm.com/asia/product/detail/W01W0101-2229.html<br><br>647 anti-goat references: https://www.thermofisher.com/antibody/product/Donkey-anti-Goat-IgG-H-L-Cross-Adsorbed-Secondary-Antibody-Polyclonal/A-21447<br><br>647 anti-chicken references: https://www.thermofisher.com/antibody/product/Goat-anti-Chicken-IgY-H-L-Secondary-Antibody-Polyclonal/A-21449<br><br>647 anti-rabbit references: https://www.thermofisher.com/antibody/product/Goat-anti-Rabbit-IgG-H-L-Cross-Adsorbed-Secondary-Antibody-Polyclonal/A-21244

Animals and other organisms

Policy information about studies involving animals; ARRIVE guidelines recommended for reporting animal research

Laboratory animals	All mice were housed with food and water available ad libitum in a 12-hour light/dark environment. Both male and female mice were used for all experiments, and mice were randomly allocated to experimental groups. For ex vivo and in vivo experiments, adult mice aged 8-12 weeks were used, unless otherwise described.<br>Aldh1L1-GFP: STOCK Tg(Aldh1l1-EGFP)OFC789Gsat/Mmucd<br>Sox9fl/fl: B6.129S7-Sox9tm2Crm/J<br>Thy1-GCaMP3: B6;CBA-Tg(Thy1-GCaMP3)6Gfng/J<br>CAG-CreER: B6.Cg-Tg(CAG-cre/Esr1*)5Amc/J
Wild animals	No wild animals were used in this study.
Field-collected samples	No field-collected samples were used in this study.
Ethics oversight	All procedures performed on mice were carried out in accordance with the ethical guidelines of the National Institutes of Health and approved by the institutional review board (IACUC Baylor College of Medicine) under protocol #AN-5596.

Note that full information on the approval of the study protocol must also be provided in the manuscript.

Flow Cytometry

Plots

Confirm that:

- [x] The axis labels state the marker and fluorochrome used (e.g. CD4-FITC).
- [x] The axis scales are clearly visible. Include numbers along axes only for bottom left plot of group (a 'group' is an analysis of identical markers).
- [x] All plots are contour plots with outliers or pseudocolor plots.
- [x] A numerical value for number of cells or percentage (with statistics) is provided.

Methodology

Sample preparation	Sox9fl/fl and Sox9+/+ mice with AAV-GFAP-iCre-P2a-TurboRFP injection in olfactory bulbs were used to purify astrocytes by fluorescence-activated cell sorting (FACS). Four weeks after the injection, olfactory bulbs from the mice were dissociated following published protocols with slight modifications. Briefly, the olfactory bulbs from 6 mice (3 control and 3 KO males) were dissected and digested for 15 min at 37°C with 0.5 mL papain solution (1x EBSS (Worthington), 230 U/ml DNase (Worthington), 25 U/ml papain (Worthington)) in 1.5mL microcentrifuge tubes on the thermomixer with 1400 rpm shacking. After digestion, the tissue was neutralized with DMEM/F12 + 10% FBS solution (ThermoFisher) and centrifuged at RT at 4200 x g for 5 min. The resultant pellet was washed by PBS and re-suspended in FACS buffer (Leibovitz's L-15 Medium with 0.01M HEPES, 0.0025% DNaseI), and filtered with tubes with cell-strainer caps (FALCON). FACS was performed in a FACSAria I (BD Bioscience, 100-μm nozzle and 20-p.s.i. setting), and astrocytes were separated by TurboRFP. FAC-sorted cells were collected in RLT lysis buffer (QIAGEN) with 1% beta-mercaptoethanol.
Instrument	FACSAria I
Software	BD FACSDiva Software
Cell population abundance	Cells were sorted directly into lysis buffer. As such purity was not confirmed by post-sorting. Purity was instead confirmed through RNAseq by cross validation with known cell-type markers.
Gating strategy	From the starting cell population, we preliminarily collected cells based on FSC-A vs SSC-A. From these cells, we collected all singlets based on FSC-A vs FSC-H. From the FSC singlets, we collected all singlets based on SSC-W vs SSC-H. A negative boundary was established through use of a wild-type mouse without AAV injection. This provides the fluorescent intensity boundaries for non-fluorescent cells. The established fluorescent boundary was confirmed with RFP+ cells.

- [x] Tick this box to confirm that a figure exemplifying the gating strategy is provided in the Supplementary Information.
